# Glycine differentially improved the growth and biochemical composition of *Synechocystis* sp. PAK13 and *Chlorella variabilis* DT025

**DOI:** 10.3389/fbioe.2023.1161911

**Published:** 2023-06-01

**Authors:** Wael A. Fathy, Hamada AbdElgawad, Ehab A. Essawy, Eman Tawfik, Mohamed S. Abdelhameed, Ola Hammouda, Shereen Magdy Korany, Khaled N. M. Elsayed

**Affiliations:** ^1^ Botany and Microbiology Department, Faculty of Science, Beni-Suef University, Beni-Suef, Egypt; ^2^ Integrated Molecular Plant Physiology Research, Department of Biology, University of Antwerp, Antwerpen, Belgium; ^3^ Biochemistry Division, Chemistry Department, Faculty of Science, Helwan University, Helwan, Egypt; ^4^ Botany and Microbiology Department, Faculty of Science, Helwan University, Helwan, Egypt; ^5^ Department of Biology, College of Science, Princess Nourah bint Abdulrahman University, Riyadh, Saudi Arabia

**Keywords:** microalgae, mixotrophic medium, glycine, biochemical composition, fatty acid profile

## Abstract

The potential of microalgae to produce valuable compounds has garnered considerable attention. However, there are various challenges that hinder their large-scale industrial utilization, such as high production costs and the complexities associated with achieving optimal growth conditions. Therefore, we investigated the effects of glycine at different concentrations on the growth and bioactive compounds production of *Synechocystis* sp. PAK13 and *Chlorella variabilis* cultivated under nitrogen availability. Glycine supplementation resulted in increased biomass and bioactive primary metabolites accumulation in both species. Sugar production, particularly glucose content, significantly improved in *Synechocystis* at 3.33 mM glycine (1.4 mg/g). This led to enhanced organic acid, particularly malic acid, and amino acids production. Glycine stress also influenced the concentration of indole-3-acetic acid, which was significantly higher in both species compared to the control. Furthermore, fatty acids content increased by 2.5-fold in *Synechocystis* and by 1.36-fold in *Chlorella*. Overall, the exogenous application of glycine is a cheap, safe, and effective approach to enhancing sustainable microalgal biomass and bioproducts production.

## Introduction

Microalgae have garnered much attention due to their ability to produce a diverse array of compounds with significant economic, medicinal, and industrial importance (Guedes et al., 2011; Santhosh et al., 2016). In this regard, microalgae have emerged as one of the most attractive and promising feedstocks for these industrial products due to their rapid growth rate, self-renewability, fast generation time, and high metabolites content (Liu et al., 2017; Fathy et al., 2021). For instance, microalgae produce astaxanthin, beta-carotene, phycocyanin, and omega-3 fatty acids (Yaakob et al., 2014; Rahman, 2020). These compounds have been shown to possess powerful antioxidant properties (Dantas et al., 2019). Recently, microalgae have been increasingly utilized in the production of cosmetic products, such as anti-aging creams and sunscreens (Yarkent et al., 2020). They also contain organic matter, which stores solar energy as biochemical energy in the form of carbon, hydrogen, oxygen, and nitrogen components (Cuellar-Bermudez et al., 2015). Despite their immense potential for a wide range of industrial products, the use of microalgae at an industrial scale is impeded by several obstacles. The high cost of production is a significant challenge, as microalgae require a controlled environment to get high biomass accumulation. Thus achieving optimal growth conditions for microalgae outdoors is a complex process (Yin et al., 2020). Consequently, the approach of combining microalgae cultivation with media containing cheap sources of nutrients (e.g., nitrogen) provided a cost-effective and eco-friendly perspective in microalgae-based bioproducts production (Nateghpour et al., 2021; Sarma et al., 2023). In this regard, enhancing the microalgal growth and bioproducts accumulation with chemicals or environmental factors had been recently used (Maity et al., 2014; Gao et al., 2023).

Nitrogen is a constituent in all structural and functional proteins such as peptides and enzymes, as well as chlorophyll, energy transfer molecules, and genetic elements in algal cells (Cai et al., 2013; Kim et al., 2016). This indeed makes nitrogen one of the most important dietary factors for algal growth. Providing enough nitrogen through the culture medium significantly speeds up cell development and enriches the biochemical content of microalgae (Wang et al., 2013; Song et al., 2023). On the other hand, nitrogen deficiency changes the organism’s metabolic route. For example, it shifted lipid metabolism away from membrane lipid production toward neutral lipid storage, resulting in the total lipid content being raised (De Bhowmick et al., 2015; Kozan et al., 2023). In this regard, most microalgae may use a range of nitrogen sources, and each source is first converted to ammonium and then metabolized into amino acids via different key metabolic enzymes (e.g., glutamine synthetase, glutamate synthase, or NADP glutamate dehydrogenase) (Cai et al., 2013; Salbitani and Carfagna, 2021). In a related study, ammonium induced a reciprocal increase in the amino acids level in *Synechocystis* sp. (Mérida et al. (1991). Through the synthesis of cyanophycin, *Synechocystis* has the remarkable capacity to internally store ammonium nitrogen (Yu et al., 2013). This nitrogen storage capacity is a promising potential for removing nitrogen from wastewater, and can be utilized as a valuable biofertilizer (Chittora et al., 2020). Increased Amino acids levels, consequently improved organic acids, and fatty acids production, where nitrogen supplementation as ammonium increased the protein content in *Dunaliella salina* (Norici et al., 2002), and lipid content in *Chlorella sorokiniana* (Wan et al., 2012). Microalgae fatty acids, for instance, are of great commercial importance in several fields, including bioenergy (Tabatabaei et al., 2011; Almomani et al., 2023). Therefore, more research is needed to increase the productivity of commercially valuable fatty acids and reduce the cost of their production. Particularly, saturated fatty acids are preferred in biodiesel production, while polyunsaturated fatty acids may have an unfavorable effect on biodiesel attributes such as ignition quality and oxidative stability (Doan et al., 2011). To this end, efficient technologies and highly productive strains were used (Maltsev and Maltseva, 2021).

Glycine, being an amino acid, contains both carbon and nitrogen that can be utilized by microalgae for many metabolic processes such as protein synthesis (De la Hoz Siegler et al., 2011; Matantseva et al., 2018). The bioavailability of glycine as a nitrogen source for microalgae is high due to its solubility and stability in the aquatic environment (Bowden et al., 2018). Glycine is readily assimilated by microalgae; thus, it can support the high growth rates and biomass production in certain microalgal species (Kim et al., 2016; Wang et al., 2016). Furthermore, glycine is a cheap and commercially available amino acid that can be easily obtained in massive quantities. Its application does not require complex and costly equipment, making it a simple and cost-effective alternative to traditional nitrogen sources such as nitrate and ammonium. This makes glycine as a potentially attractive way for large-scale microalgal cultivation.

Consequently, the cultivation of microalgae using glycine holds the potential to increase biomass production and this would also result in biomolecules accumulation including lipids, and essential amino acids. This bioproduct rich biomass has a potential as a feedstock for producing biofuel, bio fertilizers, pharmaceuticals, nutraceuticals and other bio-based products. Enhanced yield of valuable bioproducts per same microalgae biomass makes industrial production processes more efficient and profitable (Harun et al., 2010). Moreover, scaling up microalgae production on an industrial level can have cost-saving benefits, as it requires a smaller amount of cultivation space, energy, time and resources to generate the targeted level of biomolecules. These reduced production costs contribute to the increased competitiveness of microalgae-based technologies. Additionally, there is a growing market demand for products derived from microalgae. Bioactive substances such as omega-3 fatty acids, antioxidants, pigments, and biofuels are becoming more accessible and affordable (Michalak and Chojnacka, 2014; Khan et al., 2018). This accessibility has the potential to drive innovation in diverse industries including pharmaceuticals, cosmetics, and bioenergy.

The present investigation explores the impact of exogenous glycine as a nitrogenous source on the growth and bioproduct accumulation induction in the cyanobacterium *Synechocystis* sp. PAK13 and eukaryotic microalga *Chlorella variabilis*. To this end, this comprehensive study sheds light on the role of glycine in increasing the production of primary metabolites (carbohydrates, amino acids, organic acids, and lipids) and secondary metabolites such as IAA.

## Materials and methods

### Strains and cultural conditions

Microalgae strains *Synechocystis* sp*.* PAK13 and *Chlorella variabilis* DT025 were kindly provided from Algal Biotechnology Lab, Faculty of Science, Beni-Suef University, Egypt which were isolated from the marine habitat of the Red Sea and identified by using 16S and 18S rRNA gene (Supplementary Figures S1, S2). Then they were cultivated on Wuxal medium (WM), which is a universal dünger liquid plant fertilizer that has 8% N, 8% P_2_O_5_, 6% K_2_O, 0.01% B, 0.004% Cu, 0.02% Fe, 0.012% Mn and 0.004% Zn (Wilhelm Haug GmbH and Co., KG, Germany). Our strains have exhibited favorable growth in the synthetic Wuxal medium, indicating their adaptability to the employed salt concentration. To prepare the culture medium, 800 µL of WM per liter of tap water was used. To imitate the salinity of the Red Sea habitat, 1 g/L NaCl was added to the medium. This NaCl concentration was selected based on the salinity of the natural environment from which our strains were isolated (Winckelmann et al., 2015; Fathy et al., 2020). In addition to the selected NaCl concentration, we applied the optimal neutral pH 7.5 for the microalgae culture to achieve the highest yield of growth and byproducts (Wang et al., 2010). Microalgae strains were allowed to grow in the appropriate conditions, which included a temperature of 28°C ± 2°C, and light intensity was 30.4 µmolm^−2^s^−1^, a white, fluorescent light source used with a specific wavelength of 450–650 nm, and a photoperiod of 14 h light/10 h dark cycle. The temperature of microalgae cultivation was selected according to Ras et al. (2013), where the optimal temperature for our specific strains falls within the range of 28°C–30°C and they are very close to outdoors temperatures in Egypt. The photon flux density was measured using a digital lux meter (LX1330B, China). In addition, different amounts of glycine (1.66, 3.33, 6.66, 13.33, and 26.66 mM) were added to the culture mediums. These glycine concentrations were selected based on a thorough review of the literature, e.g., (Wang et al., 2016; Yue et al., 2021), and preliminary experiments conducted in our laboratory. This was also a logarithmic scale that covers a wide range of concentrations to investigate potential dose-dependent effects while maintaining a constant ratio between each concentration.

### Growth curves

In this study, a spectrophotometer (NanBei Instrument^®^, China) was used to measure the optical density of the microalgae samples at 700 nm. This allowed us to construct growth curves that provide insights into the sample’s growth dynamics as reported by Elsayed et al. (2017). To decide the wet-weight biomass of microalgae culture, we utilized a centrifugation-based approach. Specifically, we centrifuged 5 mL of our culture, then we drained the remained liquid in an inverted position on a paper tissue for 5 min at room temperature. The weight of the wet biomass was then measured. Furthermore, the duplication time, the number of generations, and the specific growth rate of our sample were measured by the established protocols by Fabregas et al. (1986) and utilizing the methodologies described by Jin et al. (2011) and Krzemińska et al. (2014). These metrics are crucial in understanding the growth characteristics of microalgae samples and were calculated to ensure the accuracy and reproducibility of the results.

## Biochemical composition investigation

### Estimation of pigment content

To quantify the photosynthetic pigments in microalgae samples, the protocol of Moran and Porath (1980) was followed. Whereas, at the exponential phase of growth, 2 mL were collected from strains culture by centrifugation at 13,000 rpm. The resulting pellets were weighed and placed in a covered glass tube, which was mixed with 5 mL of 80% ice acetone at 4°C for 24 h. After centrifugation at 13,000 rpm for 3 min, the supernatants were collected for analysis. The estimation of pigments was performed following the established protocols by Metzner et al. (1965) and Pflanz and Zude (2008), while spectrophotometric measurements were measured at 480, 645, and 663 nm.

### Estimation of soluble carbohydrates content

The quantitative estimation of soluble carbohydrates was performed by Yemm and Willis (1954). Whereas 2 mL of the exponential phase culture were centrifuged at 13.000 rpm and changed into glass-capped tubes soaked in 5 mL of absolute ethanol before boiling for 10 min in a water bath. In a test tube, 1 mL of the ethanolic extract was mixed with 4 mL of ice-concentrated anthrone reagent freshly made by dissolving 200 mg anthrone in 100 mL Conc. H_2_SO_4_ and vortexed. The tubes were then placed in a boiling water bath for 10 min before cooling in an ice bath for 5 min. Furthermore, di-saccharides were identified following van Handel (1968), whereas, to decompose reactive sucrose, 1 mL of the extract was added to 0.1 mL of 5.4 N KOH at 97°C for 10 min. The reaction mixture was then treated with 3 mL of anthrone reagent and placed in a boiling water bath for 10 min before cooling in an ice bath for 5 min. Finally, spectrophotometry was performed at 620 nm against a water reagent blank. The content of mono and di-saccharides was quantified using a standard curve of glucose and sucrose. Another standard method depends on the devices performed according to Al Jaouni et al. (2018), 2 mL microalgae was extracted in ethanol (80% v/v) by boiling for 30 s three times and once at room temperature. Derivation and resuspension of samples in dH_2_O, the supernatants were kept at 20°C for more investigation. The concentration of soluble sugars was figured out (CE in a Coulter PACE system 5500) and detected using a diode array detector. Using corresponding standards (glucose, fructose, and sucrose), concentrations were calculated.

### Estimation of organic acids content

Organic acids from microalgae strains in a mixture of 0.3% (w/v) butylated hydroxy anisole and 0.1% phosphoric acid were extracted. HPLC with a SUPELCOGEL C-610H column equipped with a UV detection system operating at 210 nm was used to estimate the concentrations of citric, succinic, fumaric, and malic acids (LaChromL-7455 diode array, LaChrom, Tokyo, Japan). Phosphoric acid (0.1% v/v) was eluted at a rate of 0.45 mL/min as the mobile phase. Oxalic, malic, succinic, citric, isobutyric, and fumaric acids were used as standards.

### Estimation of amino acids content

Amino acids were extracted from 2 mL of microalgae cultures in an aqueous ethanol concentration of 80% (v/v). The extraction buffer contained an internal standard (norvaline) to compensate for the loss of amino acids during ethanol extraction and centrifugation at 20,000 rpm for 20 min. The pellet was resuspended in chloroform after the supernatant had been evaporated. The microalgae residue was re-extracted in water (HPLC grade), and the supernatant was mixed with the chloroform-suspended pellet after centrifugation. The extracts were then centrifuged and filtered through Millipore microfilters with 0.2-μm pore size. Amino acids were separated using a BEH amide (2.1 mm × 50 mm) column and quantified using a Waters Acquity UPLC-tqd mass spectrometer (Sinha et al., 2013). A list of amino acids standards (glycine, alanine, isoleucine, leucine, methionine, valine, phenylalanine, glutamine, asparagine, threonine, serine, cystine, tyrosine, lysine, histidine, arginine, glutamic acid, and aspartate) was used as standard.

### Estimation of fatty acids

Fatty acids were extracted from 100 mg of microalgae biomass in 50% aqueous methanol at 25°C. For identification, a GC/MS analysis (Hewlett Packard 6890, MSD 5975 mass spectrometer, United States) with an HP-5 MS column (30 μm × 0.25 µm x 0.25 µm) was used, followed by fatty acid identification using the NIST 05 database and the Golm Metabolome Database (http://gmd.mpimp-golm.mpg.de) (AbdElgawad et al., 2020). A list of fatty acids standards (myristic, palmitic, heptadecanoic, stearic, arachidic, docosanoic, tricosanoic, pentacosanoic, palmitoleic, heptadecenoic, oleic, linolenic, linoleic, and eicosenoic) was used as standard.

### Statistical analysis

All trials were set up in triplicate using a completely randomized design. The data are reported as (means ± standard error) and visualized using GraphPad Prism 8.4.2 software. The Student’s t-test (*p* ≤ 0.05, *p* ≤ 0.01, *p* ≤ 0.001, and *p* ≤ 0.0001) were used to compare each experimental value was compared to the corresponding control value. A two-way ANOVA was performed (Tukey test, *p* ≤ 0.05) using the statistical package SPSS (version 20; IBM).

## Results

### Growth

**FIGURE 1:**
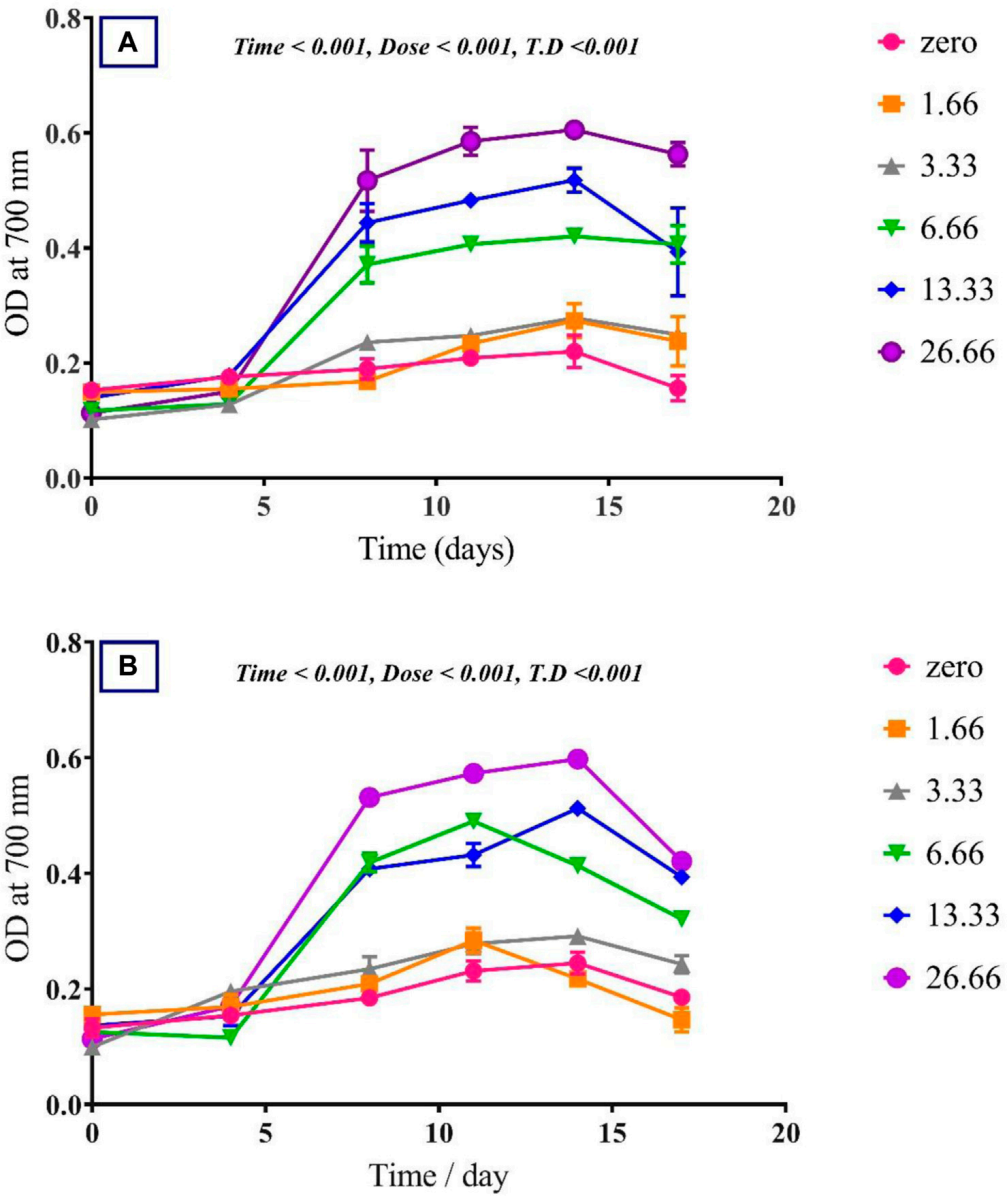
The effect of different glycine concentrations on the growth curve of **(A)** *Synechocystis* sp. and **(B)** *Chlorella* sp. Plotted points show mean daily averages ± SE, *n* = 3. The *p*-value is considered significant < 0.05 using t. test analysis.

**FIGURE 2:**
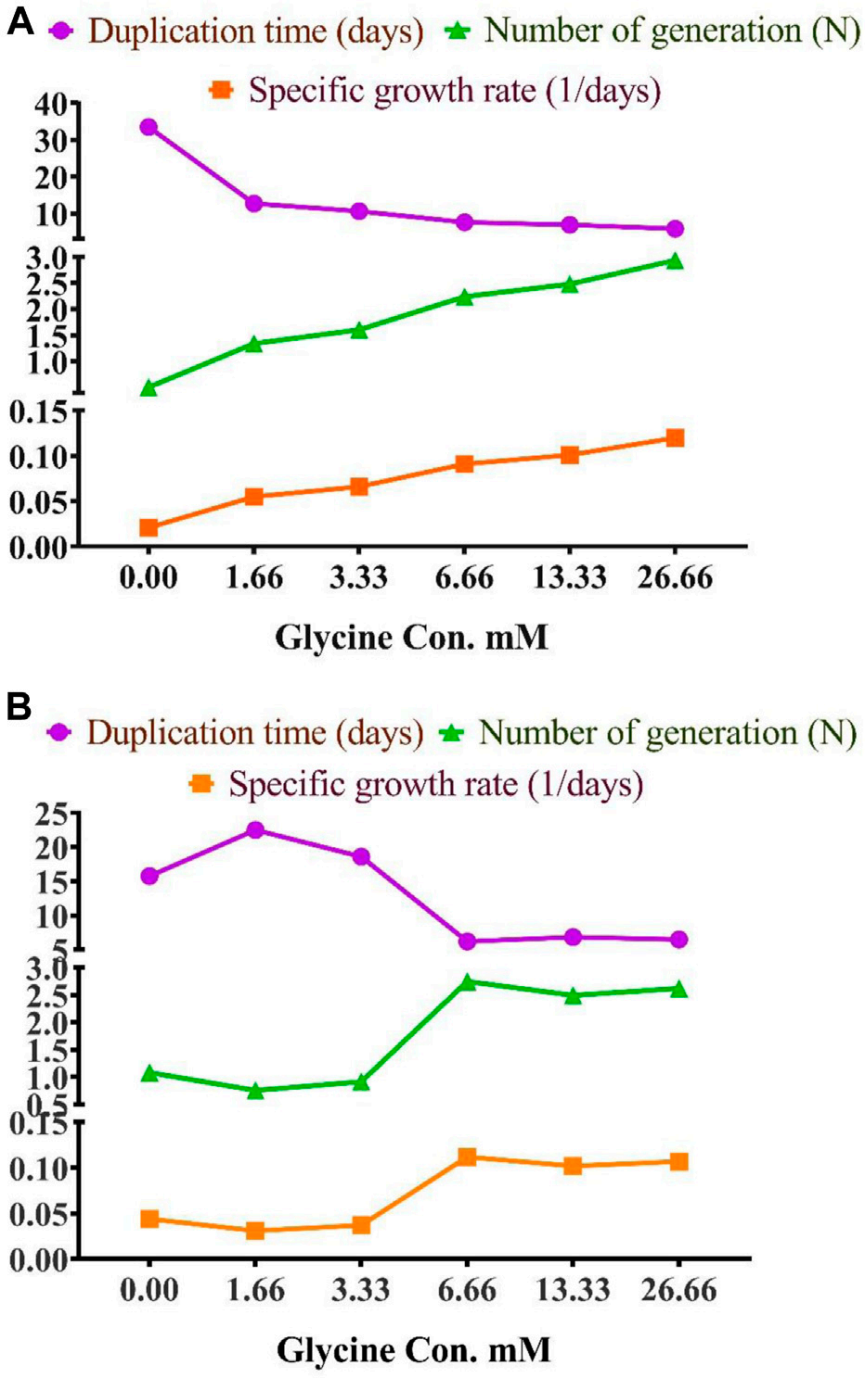
The effect of different glycine concentrations on duplication time, number of generations, and specific growth rate of **(A)** *Synechocystis* sp. and **(B)** *Chlorella* sp.

**FIGURE 3:**
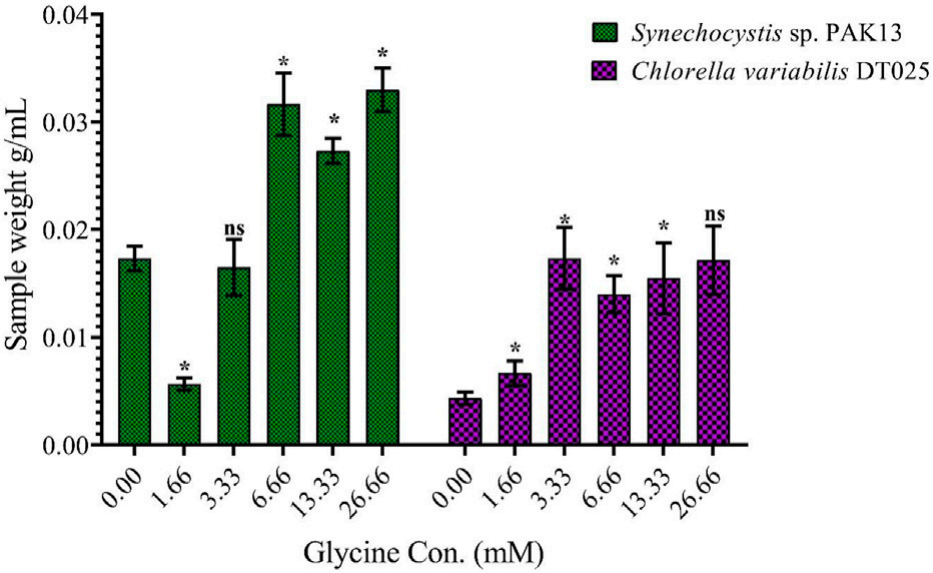
Explaining the effect of different glycine concentrations for 14 days on the wet-weight biomass of *Synechocystis* sp. and *Chlorella* sp. Data are presented as the average of three independent replicates ± SE. The statistical significances *p* > 0.05, *p* ≤ 0.05, *p* ≤ 0.01, *p* ≤ 0.001 and *p* ≤ 0.0001 were marked by the symbols ns, *, **, ***, and ****, respectively.

To assess the effect of glycine on *Synechocystis* and *Chlorella*, the growth curves of the two microalgae strains were investigated (Figure 1). The growth pattern can be divided into three distinct phases based on the growth rate, i.e., lag phase, exponential phase, and stationary phase. During the lag phase, which was seen during the initial few days, *Synechocystis* and *Chlorella* acclimatized to the new environment and the growth is slow. This phase was reflected in the data as a plateau in the OD values at time points 0–4 days. The exponential phase was characterized by a rapid increase in growth rate as the microalgae population adapts to the environment and reaches its maximum growth rate. This phase was reflected in the data as an increase in OD values at 5–14 days. The stationary phase was the period in which the microalgae population reaches it carrying capacity and the growth rate becomes stable. This phase was reflected in the data as a plateau in the OD values at 14 and 17 days. Glycine at the concentrations of 6.6, 13.3, and 26.6 mM significantly increased the growth rate of both strains. On the other hand, low doses had a slight effect on growth compared to control samples. These findings were supported (Figure 2), which showed that the duplication time decreased with increasing glycine concentrations. This increased the number of generations and specific growth rate for both strains. Furthermore, estimating biomass analysis (Figure 3) revealed that glycine concentration at 26.66 mM increased *Synechocystis* and *Chlorella* biomass production by 194% and 395% respectively, compared to the control sample. Similarly, the glycine concentrations at 6.6- and 13.3-mM glycine increased the growth by 2 folds. In contrast, 1.66 and 3.33 mM of glycine decreased the *Synechocystis* biomass compared to the control growth strain. Subsequently, it boosted both strains' growth rates, and duplication time decreased as glycine concentrations increased, resulting in a rise in the number of generations and specific growth rate for both strains. Interestingly, glycine boosted biomass production more in *Chlorella* than *Synechocystis*.

### Photosynthetic pigments

**FIGURE 4:**
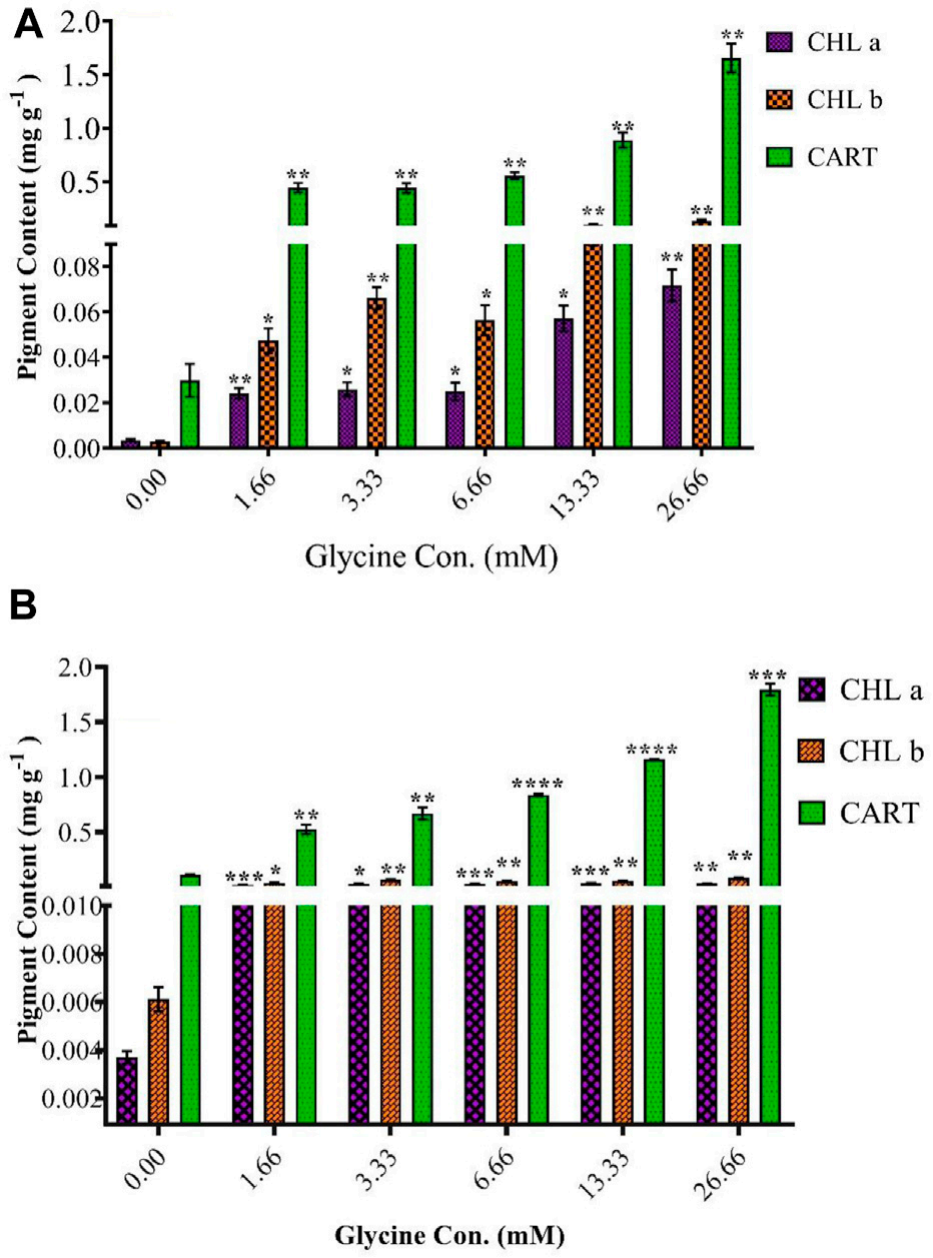
The effect of different glycine concentrations for 14 days on chlorophyll a (CHLa), chlorophyll b (CHLb), and carotenoids (CART) content in **(A)** *Synechocystis* sp. and **(B)** *Chlorella* sp. Data are presented as an average of three independent replicates ± SE. The statistical significances *p* > 0.05, *p* ≤ 0.05, *p* ≤ 0.01, *p* ≤ 0.001 and *p* ≤ 0.0001 were marked by the symbols ns, *, **, ***, and ****, respectively.

To understand the observed increases in growth, the photosynthetic pigment was estimated (Figure 4). In line with increased growth, the highest glycine concentration (26.66 mM) increased chlorophyll in Synechocystis and Chlorella by 19.4 and 9.3 folds, respectively. Similarly, chlorophyll b was induced to 44.3 and 13.7 folds at 26.66 mM in Synechocystis and Chlorella, respectively. To protect the photosynthesis system, carotenoids were significantly increased in Synechocystis by 55.3 folds, and in Chlorella by 16.3 folds higher than non-treated strain. In a nutshell, in conjunction with enhanced growth, the greatest glycine boosted chlorophyll a, b, and carotenoids in Synechocystis by 19.4, 44.3, and 55.3 folds higher than in non-treated strain, and these levels were more pronounced in Chlorella.

### Indole-3-acetic acid

The effect of glycine on microalgae cell hormone levels was demonstrated by indole-3-acetic acid (IAA) content (Table 1). IAA levels gradually increased to 2.7 folds 26.66 mM in *Synechocystis*, which was also observed in *Chlorella* increased, which showed 3.6 times increase in IAA. In summary, glycine had a positive effect on microalgal cell hormone levels.

**TABLE 1:** IAA content in *Synechocystis* sp. and *Chlorella* sp. have grown under different concentrations of glycine for 14 days.

Strain	Glycine dose	IAA mean (mg/g) ± SE
*Synechocystis* sp*.* PAK13	0	0.103 ± 0.002
	1.66	0.119 ± 0.003 ^ns^
	3.33	0.138 ± 0.004 ^ns^
	6.66	0.175 ± 0.013^*^
	13.33	0.217 ± 0.006^**^
	26.66	0.278 ± 0.013 ^ns^
*Chlorella variabilis* DT025	0	1.724 ± 0.116
	1.66	2.298 ± 0.136^*^
	3.33	3.958 ± 0.098^****^
	6.66	4.339 ± 0.123^****^
	13.33	5.250 ± 0.173^****^
	26.66	6.253 ± 0.036^****^

The statistical significances p > 0.05, p ≤ 0.05, p ≤ 0.01, p ≤ 0.001, and p ≤ 0.0001 were marked by the symbols ns, *, **, ***, and ****, respectively.

### Soluble sugar production

**FIGURE 5:**
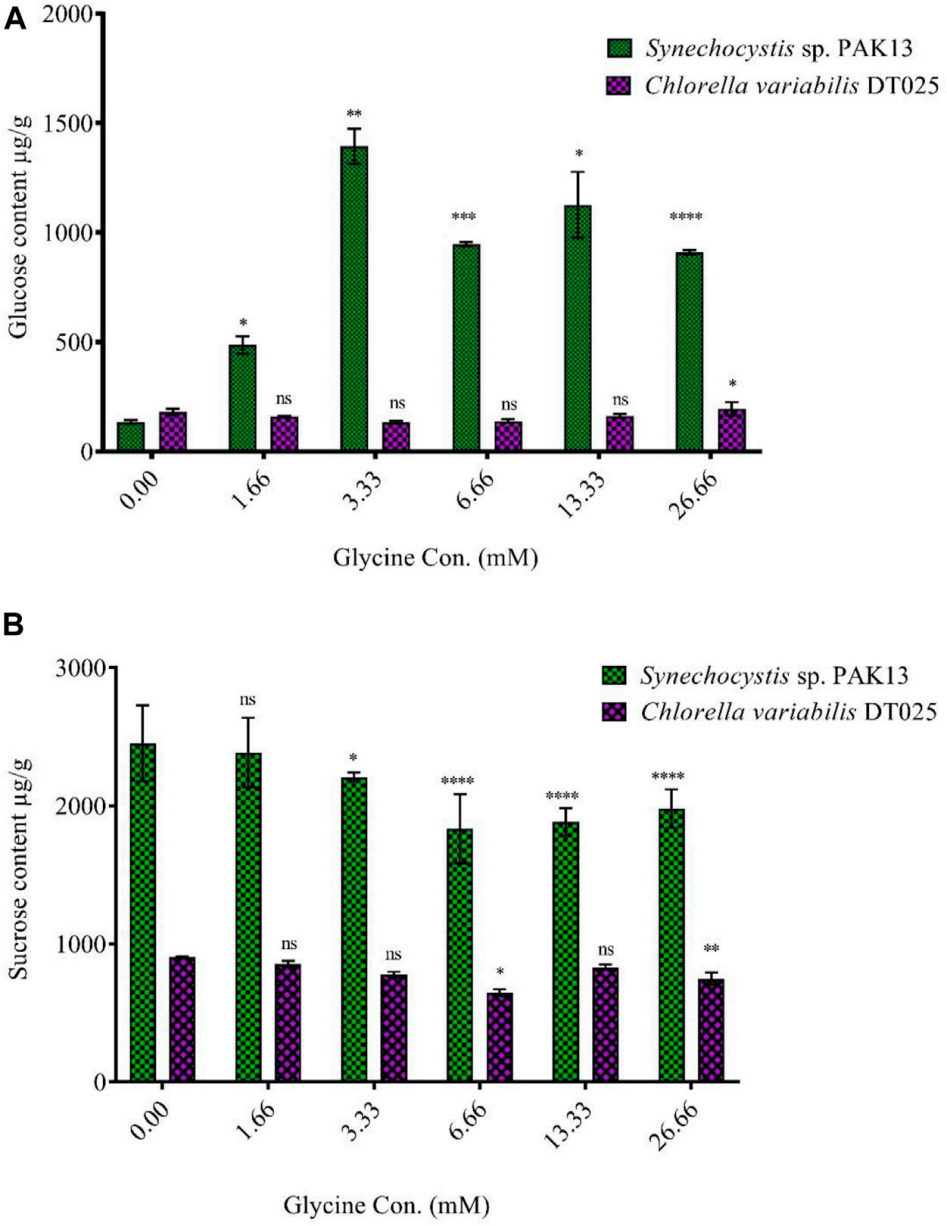
Effect of different glycine concentrations for 14 days on **(A)** glucose and **(B)** sucrose content in wet weight for *Synechocystis* sp. and *Chlorella* sp. Data are presented as the average of three independent replicates ± SE. The statistical *significances p* > 0.05, *p* ≤ 0.05, *p* ≤ 0.01, *p* ≤ 0.001 and *p* ≤ 0.0001 were marked by the symbols ns, *, **, ***, and ****, respectively.

Increased sugar production could reflect the improvement in photosynthesis under glycine exposure. Soluble sugar profiling revealed that glucose content was dramatically increased in *Synechocystis* with increasing glycine concentrations, specifically at 3.33 mM glycine (increased by 10.5 folds). On the other hand, glucose content was reduced in *Chlorella*. Moreover, sucrose levels in *Synechocystis* and *Chlorella* gradually were declined with increasing glycine concentrations at a range from 2.4 mg/g to 1.97 mg/g and 0.9 mg/g to 0.74 mg/g, respectively (Figure 5). We further measured the fructose, total soluble sugar, and glycogen content as reported in (Table 2). For *Synechocystis,* the fructose content was highest at the 1.66 glycine dose level (1.70 mg/g) and lowest in the 26.66 glycine dose level (0.66 mg/g). The total soluble sugar content was highest at the 6.66 glycine dose level (5.44 mg/g) and lowest at the control sample (3.03 mg/g). The glycogen content was highest at the 3.33 glycine dose level (88.29 mg/g) and lowest in the 26.66 glycine dose level (29.63 mg/g). While for *Chlorella,* the fructose content was highest in the 3.33 glycine dose level (1.08 mg/g) and lowest at the 1.66 glycine dose level (0.58 mg/g). The total soluble sugars content was highest in the 3.33 glycine dose level (4.00 mg/g) and lowest at the 13.33 glycine dose level (1.38 mg/g). The glycogen content was highest in the 3.33 glycine dose level (4.08 mg/g) and lowest in the control sample (3.54 mg/g). Overall, the results suggest that glycine concentration affects the fructose, total soluble sugars, and glycogen content of these two strains in different ways.

**TABLE 2:** Estimating fructose, total soluble sugars, and glycogen content in *Synechocystis* sp. and *Chlorella* sp. have grown under different concentrations of glycine for 14 days.

Strain	Glycine dose	Fructose (mg/g FW)	Total S sugars (mg/g FW)	Glycogen (mg/g FW)
		Mean ± SE	Mean ± SE	Mean ± SE
*Synechocystis* sp*.* PAK13	0	1.01 ± 0.03	3.03 ± 0.02	48.44 ± 0.36
	1.66	1.70 ± 0.03^***^	4.22 ± 0.18 ^ns^	71.10 ± 2.52^*^
	3.33	1.15 ± 0.04^*^	6.21 ± 0.37^**^	88.29 ± 4.88^ns^
	6.66	1.49 ± 0.01^*^	5.44 ± 0.15^*^	83.21 ± 1.69^**^
	13.33	0.99 ± 0.03^ns^	2.90 ± 0.05^ns^	46.72 ± 0.37^***^
	26.66	0.66 ± 0.03^***^	1.80 ± 0.08^**^	29.63 ± 0.99^*^
*Chlorella variabilis* DT025	0	0.93 ± 0.03	3.48 ± 0.18	3.54 ± 0.16
	1.66	0.58 ± 0.02^**^	3.63 ± 0.15^*^	3.37 ± 0.13^*^
	3.33	1.08 ± 0.03^***^	4.00 ± 0.29^*^	4.08 ± 0.24^ns^
	6.66	0.71 ± 0.03^*^	2.69 ± 0.17^**^	2.73 ± 0.15^**^
	13.33	0.80 ± 0.02^**^	1.38 ± 0.06^*^	1.75 ± 0.07^*^
	26.66	0.75 ± 0.02^***^	1.21 ± 0.09^*^	1.58 ± 0.08^**^

Data are presented as the average of three independent replicates ± SE. The statistical significances p > 0.05, p ≤ 0.05, p ≤ 0.01, p ≤ 0.001, and p ≤ 0.0001 were marked by the symbols ns, *, ** and ***, respectively.

### Organic acid

**FIGURE 6:**
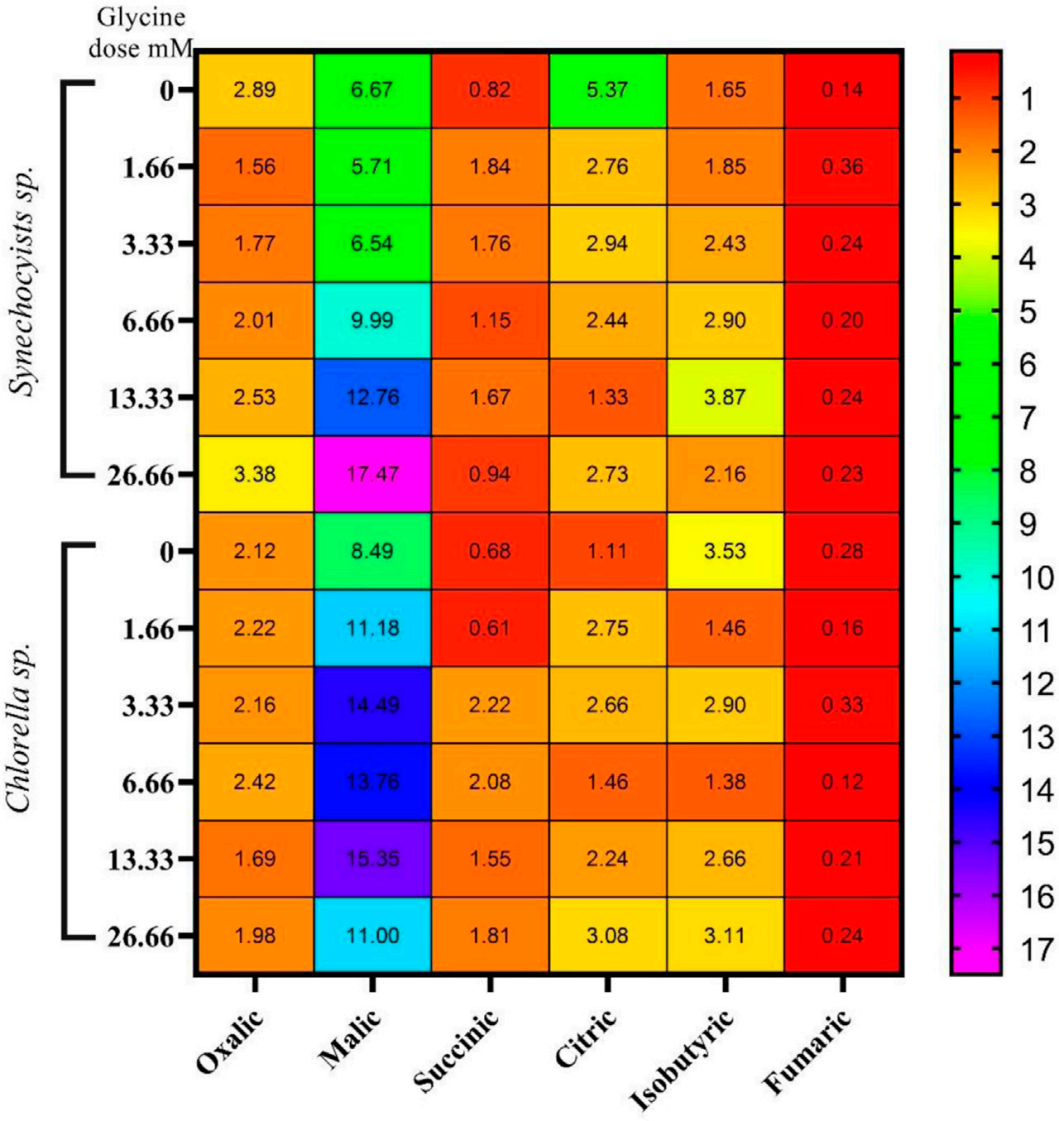
The heat map represents the effect of different glycine concentrations for 14 days on organic acids content (mg/g) wet-weight biomass *Synechocystis* sp. and *Chlorella* sp. Data are presented as the average of three independent replicates.

The changes in the levels of sugars under glycine treatment are expected to affect the tricarboxylic acid cycle intermediates, such as organic acids. Organic acids analysis indicated that they are quite different in the two strains (Figure 6) and (Supplementary Table S1). *Synechocystis* produced much higher levels of oxalic acid, citric acid, and isobutyric acid than *Chlorella*, while *Chlorella* produced higher levels of succinic acid. In *Synechocystis*, the production of oxalic acid and citric acid was significantly decreased at higher glycine doses, while the production of isobutyric acid was increased. On the other hand, the production of malic acid and fumaric acid increases at higher glycine concentrations. For *Chlorella*, the production of oxalic acid was significantly decreased at higher glycine doses, while the production of citric acid and isobutyric acid was increased. Interestingly, total organic acids concentrations were higher in *Synechocystis*. Whereas *Synechocystis* content declined in the initial dosages, after that it was gradually increased to hit 153.3% more than the control strain at a glycine dose of 26.66 mM. However, organic acid content was dramatically increased in *Chlorella* with rising glycine levels, reaching 152.7% higher than the control sample at a glycine dose of 3.33 mM. These results can be mainly attributable to malic acid. Under the most severe glycine stress, organic acid concentration was equally improved in *Synechocystis* and *Chlorella*. This is due to malic acid, which was greatly influenced by exogenous glycine. This may suggest that *Synechocystis* was more effective in organic acid metabolism and production. In conclusion, our data showed that the effect of glycine was species-specific.

### Amino acids

**FIGURE 7:**
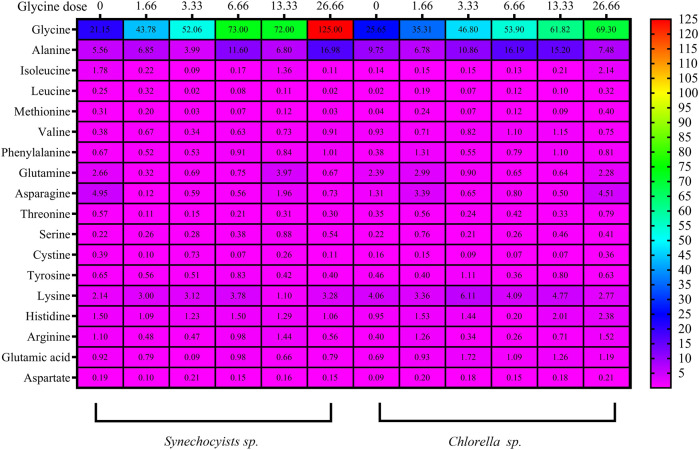
The heat map represents the effect of different glycine concentrations for 14 days on polar and nonpolar amino acids, besides acidic and basic amino acids content (mg/g) in *Synechocystis* sp. and *Chlorella* sp. Data are presented as the average of three independent replicates.

The impact of glycine provision on the amino acid profiles of the two strains was assessed. Glycine altered the levels of both nonpolar and polar amino acids. The concentrations of the nonpolar amino acidswere gradually increased with rising glycine concentration (Figure 7; Supplemenatry Table S2). Under 26.6 mM, nonpolar amino acids were increased to hit 4.8 folds in *Synechocystis* glycine compared to those in the non-treated strain. While *Chlorella* showed an increase of 2.2 folds higher than the control strain under 26.6 mM glycine. Consequently, it was obvious from the table interpretation that glycine was the main nonpolar amino acid affected by glycine exposure. Where 70% of the nonpolar amino acids content, specifically in *Synechocystis* was increased by 5.93 folds higher than the control one at the dose of 26.66 mM glycine. Overall, it can be observed that both *Synechocystis* and *Chlorella* had an increased nonpolar amino acids content with increasing glycine concentration. This trend is observed for most tested amino acids, including alanine, isoleucine, leucine, methionine and valine. Interestingly, *Synechocystis* had a much larger increase in nonpolar amino acid content with increasing glycine concentration. For example, at a glycine concentration of 26.66 mM, *Synechocystis* had a total nonpolar amino acid content of 144.47 mg/g, while *Chlorella* had a total nonpolar amino acid content of only 81.20 mg/g. Phenylalanine content also showed a different pattern, *Synechocystis* had a slight increase in phenylalanine content, while *Chlorella* showed a more significant increase in phenylalanine content at 13.33 mM and 26.66 mM. On the other hand, polar amino acids in *Synechocystis* (Figure 7) and (Supplementary Table S3) were significantly declined under all glycine dosages. For instance, at 26.66 mM, glycine was reduced by 3.5 folds compared to the control strain. Moreover, polar amino acids were decreased in *Chlorella*, with the most glycine doses around half the control one, except for 1.6- and 26.6-mM glycine. This suggested that the polar amino acids content in both species varies depending on the concentration of glycine. For example, in *Synechocystis*, the total polar amino acids content was decreased from 9.43 at the control to 7.79 at 13.33 mM glycine and it was decreased to 2.751 at 26.66 mM glycine. While, in *Chlorella*, the total polar amino acids content was steadily increased from 4.89 at control to 8.97 at 26.66 mM glycine. The differences in the specific polar amino acid content between the two species. For example, at control conditions, *Synechocystis* had a higher content of glutamine, asparagine, and tyrosine, while *Chlorella* had a higher content of threonine, serine and cysteine. However, as the glycine concentration was increased, the differences between the two species became less pronounced.

Furthermore, basic and acidic amino acid analyses revealed a steady state under all glycine treatments, while the greatest basic amino acid concentration in *Synechocystis* was 1.3 folds greater at 6.6 mM glycine (Figure 7; Supplementary Table 4). However, this was slightly different, but it was 1.5 folds higher at 3.3 mM glycine in *Chlorella*. The basic amino acids content was generally increased, except for lysine at the highest dose of 13.33, where it was significantly decreases. The acidic amino acids content, on the other hand, showed no consistent trend across the different glycine doses. For *Chlorella*, the results showed that the increased glycine dose, generally increased the basic amino acids content, except for histidine at 26.66 mM, where it was decreased significantly. The acidic amino acid content also showed no consistent trend across the different glycine doses.

### Fatty acids

The induced effects of glycine could also be extended to include the tricarboxylic acid cycle intermediates, such as fatty acids. Thus, we measured the individual and total fatty acids (saturated and unsaturated), to get an overview of their potential for biodiesel production. To demonstrate these changes, we measured saturated fatty acids levels (Table 3). SFA levels were steadily increased, whereas SFA levels in *Synechocystis* reached 247% more than the control sample at 26.66 mM, and this was also observed in *Chlorella* (242% increase compared to control).

**TABLE 3:** Estimating saturated fatty acids content (mg/g) in *Synechocystis* sp. and *Chlorella* sp. have grown under different concentrations of glycine for 14 days.

Strain	Glycine dose	Myristic (C14:0)	Palmitic (C16:0)	Heptadecanoic (C17:0)	Stearic (C18:0)	Arachidic (C20:0)	Docosanoic (C22:0)	Tricosanoic (C23:0)	Pentacosanoic (C25:0)	Sum of saturated FA ± SE
		Mean ± SE	Mean ± SE	Mean ± SE	Mean ± SE	Mean ± SE	Mean ± SE	Mean ± SE	Mean ± SE	
*Synechocystis* sp. PAK13	0	0.332 ± 0.005	15.193 ± 0.333	0.025 ± 0.001	1.393 ± 0.092	1.136 ± 0.013	0.609 ± 0.020	0.020 ± 0.002	0.002 ± 0.0002	18.71 ± 0.47
	1.66	0.366 ± 0.002 ^ns^	17.590 ± 0.507 ^ns^	0.024 ± 0.001^ns^	1.421 ± 0.079 ^ns^	1.041 ± 0.020 ^ns^	0.486 ± 0.022 ^ns^	0.020 ± 0.002 ^ns^	0.002 ± 0.0002 ^ns^	20.95 ± 0.63
	3.33	0.564 ± 0.001^**^	20.286 ± 0.656 ^ns^	0.032 ± 0.001^ns^	1.778 ± 0.095 ^ns^	0.725 ± 0.016^**^	0.404 ± 0.083 ^ns^	0.026 ± 0.003 ^ns^	0.003 ± 0.0003 ^ns^	23.82 ± 0.86
	6.66	0.672 ± 0.012^**^	25.701 ± 1.960 ^ns^	0.047 ± 0.002^ns^	2.591 ± 0.092^*^	1.613 ± 0.150 ^ns^	0.531 ± 0.056 ^ns^	0.037 ± 0.003 ^ns^	0.002 ± 0.0003 ^ns^	31.19 ± 2.28
	13.33	0.772 ± 0.004^****^	32.081 ± 0.975^*^	0.067 ± 0.002^*^	2.014 ± 0.112 ^ns^	1.799 ± 0.015^***^	0.406 ± 0.019^*^	0.047 ± 0.005 ^ns^	0.005 ± 0.0005 ^ns^	37.19 ± 1.13
	26.66	0.870 ± 0.019^**^	41.207 ± 2.038^*^	0.088 ± 0.002^**^	1.426 ± 0.140 ^ns^	2.124 ± 0.183 ^ns^	0.433 ± 0.012 ^ns^	0.045 ± 0.013 ^ns^	0.007 ± 0.0008 ^ns^	46.20 ± 2.41
*Chlorella variabilis* DT025	0	0.313 ± 0.003	16.254 ± 0.597	0.061 ± 0.003	1.262 ± 0.054	1.225 ± 0.043	0.462 ± 0.077	0.040 ± 0.005	0.004 ± 0.0005	19.62 ± 0.78
	1.66	0.307 ± 0.005 ^ns^	17.541 ± 0.733 ^ns^	0.028 ± 0.001^*^	1.099 ± 0.056 ^ns^	1.033 ± 0.029 ^ns^	0.455 ± 0.024 ^ns^	0.021 ± 0.002 ^ns^	0.002 ± 0.0002 ^ns^	20.49 ± 0.85
	3.33	0.572 ± 0.024 ^ns^	24.133 ± 1.625 ^ns^	0.084 ± 0.006 ^ns^	1.094 ± 0.027 ^ns^	1.370 ± 0.102 ^ns^	0.680 ± 0.071 ^ns^	0.036 ± 0.005 ^ns^	0.004 ± 0.0005 ^ns^	27.97 ± 1.86
	6.66	0.912 ± 0.008^***^	28.813 ± 0.806^**^	0.098 ± 0.004 ^ns^	2.301 ± 0.098^*^	0.924 ± 0.032 ^ns^	0.610 ± 0.039 ^ns^	0.066 ± 0.008 ^ns^	0.007 ± 0.0008 ^ns^	33.73 ± 1.00
	13.33	0.733 ± 0.004^****^	31.220 ± 1.012^*^	0.072 ± 0.002 ^ns^	2.219 ± 0.114 ^ns^	1.597 ± 0.179 ^ns^	1.018 ± 0.041 ^ns^	0.037 ± 0.002 ^ns^	0.004 ± 0.0004 ^ns^	36.90 ± 1.35
	26.66	0.554 ± 0.014^*^	40.354 ± 0.904^**^	0.045 ± 0.001 ^ns^	2.822 ± 0.070^**^	2.152 ± 0.390 ^ns^	1.424 ± 0.048^*^	0.023 ± 0.002 ^ns^	0.004 ± 0.0009 ^ns^	47.38 ± 1.43

Data are presented as the average of three independent replicates ± SE. The statistical significances p > 0.05, p ≤ 0.05, p ≤ 0.01, p ≤ 0.001, and p ≤ 0.0001 were marked by the symbols ^ns^, *, ** and ***, ****, respectively.

The results showed that the total SFA content increased with increasing glycine concentration in both microalgal species. For instance, the total SFA in *Synechocystis* was increased from 18.71 to 46.20 mg/g, at 26.66 mM. While in *Chlorella* increased from 19.62 to 33.73 mg/g, over the same range of glycine concentrations. This indicated that glycine positively influenced the SFA synthesis in these microalgae. Regarding individual SFA, the results showed that the two microalgal species showed different types and amounts of FA produced in response to glycine. In *Synechocystis*, the major produced SFAs were palmitic (C16:0) and myristic (C14:0) acids. However, the content of heptadecanoic (C17:0) and stearic (C18:0) acids did not show significant changes, while arachidic (C20:0), docosanoic (C22:0), tricosanoic (C23:0), and pentacosanoic (C25:0) acids were significantly increased only at 6.66 and 13.33 mM. In contrast, in *Chlorella*, the major produced SFAs were palmitic (C16:0) and stearic (C18:0) acids. The content of myristic (C14:0) acid did not show significant changes, but heptadecanoic (C17:0), arachidic (C20:0), docosanoic (C22:0), tricosanoic (C23:0), and pentacosanoic (C25:0) acids were significantly increased only 13.33 mM. Overall, the results suggested that glycine supplementation differentially enhanced the production of SFA in both species.

Furthermore, unsaturated fatty acids (UFA) demonstrate that at a dose of 26.66 mM glycine, UFA in *Synechocystis* was increased to 250% greater than the control strain (Table 4). At 26.66 Mm, the UFA in *Chlorella* increased by 136%. In *Synechocystis* 26.66 mM of glycine led to the highest total fatty acid content (174.14 mg/g). The content of UFA at the highest dose led to the highest content of UFAs (127.943 mg/g). Among the UFAs, oleic acid (C18:1) showed the highest content in all samples, ranging from 27.631 to 72.671 mg/g. The other UFAs showed a slight increase, with linolenic acid (C18:3) of the most significant increase. While in *Chlorella* the total fatty acids content was also increased. At 26.66 mM lead to the highest total fatty acids content (118.54 mg/g). At UFAs levels, the content of palmitoleic acid (C16:1) was decreased, while the content of heptadecenoic acid (C17:1) showed a slight increase. Oleic acid (C18:1) showed a slight decrease, while linolenic acid (C18:3) and linoleic acid (C18:2) showed an increase. Overall, our findings suggested that glycine-treated *Chlorella* or *Synechocystis* cultivation can be used to produce biofuels and other bioproducts.

**TABLE 4:** Estimating unsaturated fatty acids and the total amount of fatty acids content (mg/g) in *Synechocystis* sp. and *Chlorella* sp. have grown under different concentrations of glycine for 14 days.

Strain	Dose	Palmitoleic (C16:1)	Heptadecenoic (C17:1)	Oleic (C18:1)	Linolenic (C18:3)	Linoleic (C18:2)	Eicosenoic (C20:1)	Sum of unsaturated FA ± SE	Total FA
		Mean ± SE	Mean ± SE	Mean ± SE	Mean ± SE	Mean ± SE	Mean ± SE		
*Synechocystis* sp*.* PAK13	0	0.06 ± 0.008	0.138 ± 0.013	27.631 ± 1.424	4.719 ± 0.156	17.780 ± 0.879	0.876 ± 0.038	51.208 ± 2.517	69.92 ± 2.98
	1.66	0.06 ± 0.007 ^ns^	0.136 ± 0.013 ^ns^	30.253 ± 1.039 ^ns^	6.184 ± 0.303 ^ns^	19.703 ± 1.438 ^ns^	0.772 ± 0.056 ^ns^	57.110 ± 2.857	78.06 ± 3.49
	3.33	0.06 ± 0.011 ^ns^	0.123 ± 0.012 ^ns^	41.040 ± 1.004^*^	7.325 ± 0.201^*^	21.082 ± 1.032 ^ns^	0.841 ± 0.038 ^ns^	70.478 ± 2.298	94.30 ± 3.15
	6.66	0.082 ± 0.007^*^	0.183 ± 0.019 ^ns^	47.114 ± 0.387^*^	8.381 ± 0.525 ^ns^	25.904 ± 2.137 ^ns^	1.013 ± 0.084 ^ns^	82.678 ± 3.159	113.87 ± 5.44
	13.33	0.124 ± 0.014^**^	0.167 ± 0.016 ^ns^	49.564 ± 2.531 ^ns^	8.410 ± 0.399 ^ns^	32.773 ± 2.442 ^ns^	1.323 ± 0.097 ^ns^	92.362 ± 5.499	129.55 ± 6.63
	26.66	0.166 ± 0.021 ^ns^	0.150 ± 0.014 ^ns^	72.671 ± 1.675^**^	14.651 ± 0.834^*^	38.714 ± 4.579 ^ns^	1.592 ± 0.191 ^ns^	127.943 ± 7.313	174.14 ± 9.72
*Chlorella variabilis* DT025	0	0.093 ± 0.004	0.148 ± 0.015	33.155 ± 1.589	7.239 ± 0.285	25.670 ± 0.615	1.023 ± 0.020	67.328 ± 2.527	86.95 ± 3.31
	1.66	0.070 ± 0.006^*^	0.134 ± 0.008 ^ns^	32.987 ± 0.711 ^ns^	6.042 ± 0.349 ^ns^	21.021 ± 1.208^*^	0.832 ± 0.048 ^ns^	61.086 ± 2.331	81.57 ± 3.18
	3.33	0.111 ± 0.006 ^ns^	0.150 ± 0.019 ^ns^	46.128 ± 1.446^***^	6.968 ± 0.510 ^ns^	24.407 ± 1.403^*^	0.968 ± 0.050 ^ns^	78.732 ± 3.434	106.70 ± 5.29
	6.66	0.206 ± 0.020 ^ns^	0.128 ± 0.013 ^ns^	50.098 ± 3.236^*^	6.422 ± 0.466^*^	23.040 ± 1.526 ^ns^	0.923 ± 0.059 ^ns^	80.818 ± 5.319	114.55 ± 6.32
	13.33	0.109 ± 0.003^***^	0.197 ± 0.017 ^ns^	35.187 ± 1.463^**^	9.243 ± 0.666 ^ns^	29.286 ± 1.998^*^	1.292 ± 0.089 ^ns^	75.315 ± 4.236	112.21 ± 5.59
	26.66	0.040 ± 0.009^**^	0.267 ± 0.021 ^ns^	20.011 ± 0.481 ^ns^	12.060 ± 0.878^**^	37.122 ± 1.469^**^	1.660 ± 0.119 ^ns^	71.159 ± 2.978	118.54 ± 4.41

Data are presented as the average of three independent replicates ± SE. The statistical significances p > 0.05, p ≤ 0.05, p ≤ 0.01, p ≤ 0.001, and p ≤ 0.0001 were marked by the symbols ^ns^, *, ** and ***, ****, respectively.

## Discussion

### Glycine improved biomass accumulation

The biomass results revealed that glycine at 6.6- and 13.3 and/or 26.66 mM increased biomass production in *Synechocystis* and *Chlorella*, compared to the control sample. In contrast, 1.66 and 3.33 mM of glycine decreased *Synechocystis* biomass compared to the control growth strain. To understand this increase in the biomass accumulation, the growth curves of both *Synechocystis* and *Chlorella* strains were analyzed. During the lag phase, the microalgae acclimatized to the new environment, resulting in slow growth. The exponential phase was characterized by a rapid increase in growth rate. The results of the present study showed that glycine significantly increased the growth rate of both strains, particularly at 6.6, 13.3, and 26.6 mM. The low concentration of glycine had a slight effect on growth compared to control samples. These findings were supported by the results of glycine concentration, which showed that the duplication time decreased with increasing glycine concentrations, increasing the number of generations and specific growth rate for both strains.

Furthermore, at high concentrations glycine showed the fastest cell growth rate. These findings were previously validated by Kumar et al. (2014) who reported that glycine treatment increased *Chlorella* cell production, furthermore, both biomass and growth rate were much improved. The highest concentration was higher (18.95%) than the control value (Garcıa et al., 2005). Similar to our results, Wang et al. (2016) found that *Chlorella sorokiniana* dry weight increased in a dose-dependent manner with biomass to 121% compared to the control. We hypothesized, that glycine boosts biomass and biomolecule productivity by stabilizing and improving the C4 system (Sun et al. (2016). Thus, enhancing photosynthetic efficiency allowed strains to use more solar energy when treated with glycine, this added energy was subsequently converted into more biomass and lipids. Similarly, mixotrophic cultures experience a rise in pigments that was proportional to the increase in biomass concentration (Camacho et al., 1999). Briefly, the increase in antioxidant carotenoids played a crucial role in the regulation of oxidative stress in microalgae, preventing oxidative damage to algal cells. Moreover, one of the most common antioxidants was glycine, which had been used to boost microalgae lipid production (Song et al., 2022).

Moreover, this study proved that glycine treatment increased the concentration of IAA, a plant hormone that promotes growth and development. The concentration of IAA was significantly higher in both strains at the highest glycine concentration. Glycine had a favorable effect on microalgae cell hormone (IAA) levels in *Chlorella*, which was higher than *Synechocystis*. Low dosages of IAA enhanced the cell proliferation by triggering genes that promote cell division (González-Garcinuño et al., 2016). It was reported before that, hormones such as IAA, gibberellic acid, and cytokinin-kinetin have a role in increasing the biomass percentages of *Chlorella sorokiniana* (Guldhe et al., 2019).

### Glycine treatment directed algal metabolism toward fatty acid production

In this regard, microalgae prefer glycine as an organic nitrogen source because the metabolic cost is lower than the cost of other nitrogen forms (Mandal et al., 2018). Most microalgae may employ a variety of nitrogen sources, and each source is first converted to ammonium and then metabolized into amino acids via various mechanisms such as glutamine synthetase, glutamate synthase, or NADP glutamate dehydrogenase (Lasa et al., 2002). Moreover, glycine induced an increase in photosynthesis that induces sugar production. Sugars could supply building blocks for the biosynthesis of fatty acids, which are a part of the tricarboxylic acid cycle intermediates. Consistently, the total soluble sugar level increased at low glycine concentrations, but it decreased at the highest concentration, particularly in *Chlorella*. This decrease may indicate the shift of metabolism toward other primary or secondary metabolites. The results showed that organic acid concentration improved equally in *Synechocystis* and *Chlorella*. As a result, this sequence enhanced the generation of organic acids and fatty acids (Cai et al., 2013; Salbitani and Carfagna, 2021). Total fatty acids during our study in *Synechocystis* and *Chlorella* significantly boosted compared to the control sample. In agreement, Wang et al. (2016) indicated that *Chlorella* lipid yield was also upregulated by exogenous glycine treatment at a concentration of 500 mg/L increasing lipid production to 138%. In a previous study by Zhao et al. (2016), photo-chemical modulation enhanced lipid content in *Monoraphidium* sp. The study reported the largest lipid content of 48.5%, which represented a significant improvement in lipid accumulation compared to baseline levels. One of these enhancements, membrane lipids contain the majority of unsaturated fatty acids, and their primary role was to maintain membrane fluidity under various situations (Zhila et al., 2011).

### Glycine-treated *Synechocystis* and *Chlorella* are promising sources of bioactive compounds

Microalgae have emerged as a potential feedstock for bioactive compound production due to their high content of essential and unsaturated fatty acids. Chemical environmental modulations can alter microalgae metabolic pathways to accumulate high amounts of neutral lipids from 20–50 percent dry cell weight, primarily in the form of TAGs, in addition to other compounds such as carbohydrates and secondary metabolites (Elsayed et al., 2017). Various methods, such as chemical treatments, growth environment modifications, and genetic engineering, have been employed to induce bioactive primary metabolite production in microalgae (Goh et al., 2019). Whereas researchers aimed to identify microalgae strains capable of high lipid synthesis under stressful conditions. They specifically investigated the lipid content of *Chlorella* and *Synechocystis* under temperature stress. Their findings revealed that *Chlorella* had a lipid content of 6.81E-13 g/cell, while *Synechocystis* had a slightly higher lipid content of 8.19E-13 g/cell. These studies further supported and confirmed our own results, indicating that cyanobacterium cells accumulated more lipids than chlorophyta cells under glycine stress conditions (Elsayed et al., 2017). Whereas, our findings showed that under glycine stress conditions, cyanobacterium cells (*Synechocystis*) accumulated more lipids than chlorophyta cells (*Chlorella*). Glycine-treated *Synechocystis* and *Chlorella* are therefore promising feedstocks for biofuel production due to their high lipid accumulation potential.

On utilizing the microalgae on a large industrial scale, we cannot deny the role of pH and temperature on microalgae growth and metabolism, whereas pH strongly affects microalgae enzymatic activities and metabolic pathways. In this context, pH variations had significant implications for biomolecule production as they can either enhance or hinder microbial metabolism (Sassenhagen et al., 2015). While acidic pH conditions can have a detrimental effect on microbial metabolism. Enzyme activity can be inhibited, cellular processes disrupted and metabolic pathways impaired under low pH values (Lund et al., 2014). When NH_4_Cl was added to *Synechocystis* cells that were utilizing nitrate as their nitrogen source, there was a rapid decline in growth and cell activity. However, by adjusting the pH and transferring the cells to a medium without ammonium, the initial activity was fully restored after washing the cells (Kolodny et al., 2006). On the other hand, high pH levels can disrupt enzyme stability and function, leading to reduced metabolic activity (Frankenberger Jr and Johanson, 1982). Thus, here to achieve the highest yield of growth and byproducts, we employed the optimal neutral pH for the microalgae culture as reported before by Wang et al. (2010).

Similar to pH, environmental conditions, such as temperature showed a notable impact on the biochemicals production of microalgae. Elevated temperatures during growth had been associated with a significant decrease in protein content, accompanied by increases in lipids and carbohydrates (Renaud et al., 2002). Moreover, different studies have shown that the response of microalgal chemical composition to high and low growth temperatures varies across species. The ideal growth temperatures are those that enable the cells to undergo photosynthesis without altering their inherent biochemical or physiological characteristics (Ras et al., 2013). In the context of Egypt, where microalgae are cultivated outdoors, they must thrive within a wide diurnal temperature range of 25°C–35°C consistently throughout the year. According to Ras et al. (2013) and our preliminary results, the optimal temperature for our specific strains falls within the range of 28°C–30°C.

Overall, our study contributes to the growing body of research on microalgae as a potential feedstock for biofuel production. Additionally, glycine can serve as both a carbon and nitrogen source, making it useful for promoting the heterotrophic growth of microalgae under stressful conditions and increasing biomass production (Yu et al., 2021). Glycine can then be utilized in various metabolic pathways within the cell, such as protein synthesis and photorespiration (Wingler et al., 2000; Ren et al., 2022). In protein synthesis, glycine can serve as one of the building blocks of proteins and can be directly incorporated into the growing polypeptide chain during translation. In photorespiration, glycine is converted into serine with the release of carbon dioxide and ammonia (Keys, 2006).

### Glycine uptake can explain the differential response of *Synechocystis* and *Chlorella* responded to glycine treatment

Glycine treatment positively changed the growth and biomass besides IAA, organic acid, and amino acids and decreased total soluble sugars in *Synechocystis*. However, glycine treatment did not significantly affect any notable change in total FA and total soluble sugar levels. Overall, *Synechocystis* shows a more pronounced effect on various metabolites. While *Chlorella* shows a more specific effect on amino acids and IAA production. Specific metabolic pathways and physiological characteristics of these two microalgal strains could also play a role in differential response to glycine. The microalgae can uptake the glycine using a transport system that enables direct transportation into the cell, where it can serve as a nitrogen source for the cell (Yu et al., 2021). Here the difference in the rate of glycine uptake between prokaryotic *Synechocystis* and eukaryotic *Chlorella* cells may contribute to their different responses to glycine (Gao et al., 2009; Kanwal and De-Eknamkul, 2023). In this regard, some microalgae species have been shown to preferentially utilize glycine over other nitrogen sources such as ammonium and nitrate (Flynn and Butler, 1986; Tyler et al., 2005).

Our growth WM medium contains nitrogen in forms that could potentially affect glycine uptake. In this regard, the study of Gopalakrishnan et al. (2015) that used glycine as a nitrogen source revealed that the majority of the glycine and serine detected in the *Chlorella protothecoides* intracellular were unlabeled, indicating that these amino acids were primarily taken up from the extracellular medium. Interestingly, nitrogen-limited growth conditions decreased glycine uptake, indicating that nitrogen availability in the growth media improved glycine uptake (Gopalakrishnan et al., 2015).

**FIGURE 8:**
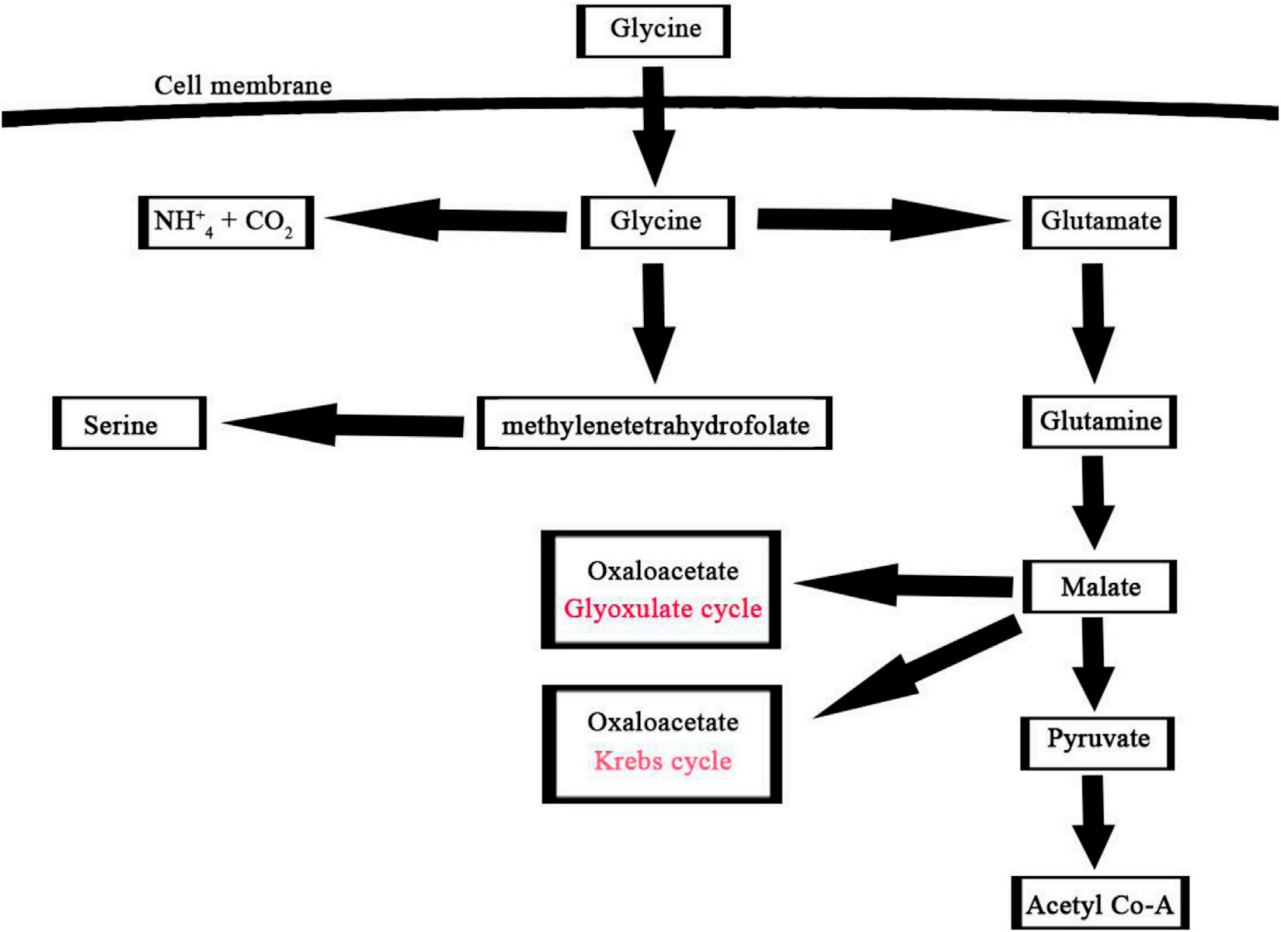
Glycine assimilation pathway in microalgae, this pathway illustrates the main three steps involved in the utilization of glycine by microalgae for metabolic processes such as glutamine, methylenetetrahydrofolate, and ammonium pathways.

A noteworthy consequence of using glycine as the nitrogen source was the partial activation of the glyoxylate shunt. Therefore, glycine provided in the extracellular growth medium as a nitrogen source can either be catabolized through glycine dehydrogenase for amino acids production or directed into the glyoxylate shunt for TCA cycle intermediates as shown in (Figure 8) (Gopalakrishnan et al., 2015). Overall, algal metabolism utilizing glycine highlights the significant impact that the nature of the nitrogen source can have on the labeling patterns of intracellular metabolites and metabolite flows.

## Conclusion

**FIGURE 9:**
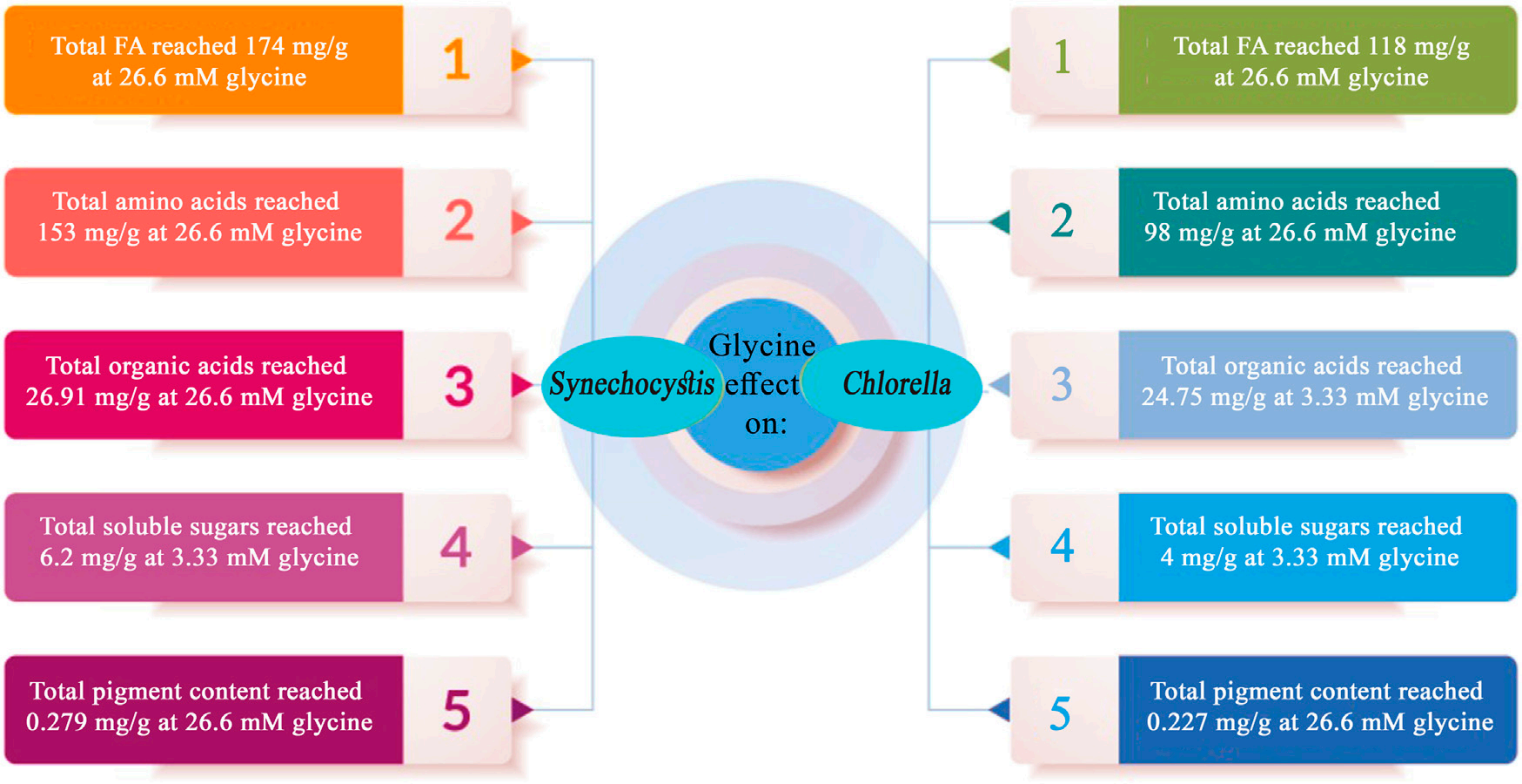
Summary of the key findings obtained through the application of external glycine on *Synechocystis* sp. and *Chlorella* sp.

In a nutshell, the present study explored the effect of glycine on two microalgal strains, *Synechocystis* and *Chlorella*, and found that glycine had a positive impact on their growth, photosynthetic pigments, cell hormone levels, organic acid production, amino acid and fatty acid synthesis in microalgae as presented in (Figure 9). Glycine also had a positive effect on the levels of the microalgae cell hormone indole-3-acetic acid. Total soluble sugar was lowered in both strains, but to a greater extent in *Chlorella*. This study demonstrated the impact of glycine supplementation on the amino acid and fatty acid profiles of *Synechocystis* and *Chlorella*. The response to glycine concentration differs between the two strains, with *Chlorella* showing a significant increase in phenylalanine content at higher glycine concentrations. The findings suggest that glycine supplementation can enhance the production of nonpolar amino acids and potentially increase the potential for bioactive compounds and biodiesel production by altering the fatty acid profiles of these strains. The findings presented in this article have wide-ranging ramifications for the industry. Whereas this information is valuable for microalgae-cultivating industries because it provides insight into the potential use of glycine as a growth stimulant. In addition, the results suggest that glycine supplementation can modify the fatty acid profiles of microalgal strains, thereby potentially boosting the production of bioactive compounds and biodiesel. This has implications for industries focused on biofuel production and those investigating microalgae as a sustainable dietary source. Overall, the study emphasizes the promising potential of glycine supplementation in large-scale microalgae cultivation as a possible alternative to conventional crops for food and biofuel production.

## Data Availability

The original contributions presented in the study are included in the article/Supplementary Material, further inquiries can be directed to the corresponding author.
