# Supplementary material for: Glycine differentially improved the growth and biochemical composition of Synechocystis sp. PAK13 and Chlorella variabilis DT025
Source: Front Bioeng Biotechnol. 2023 Jun 1;11:1161911. doi: 10.3389/fbioe.2023.1161911 (PMC10267400; doi:10.3389/fbioe.2023.1161911)

Table 1. Different organic acids content (mg/g) in *Synechocystis* sp. and *Chlorella* sp. grown under different concentrations of glycine for 14 days.

| Strain | Glycine Dose | Oxalic | Malic | Succinic | Citric | Isobutyric | Fumaric | Sum of organic acid (mg/g) |
|---|---|---|---|---|---|---|---|---|
| | | Mean ± SE | Mean ± SE | Mean ± SE | Mean ± SE | Mean ± SE | Mean ± SE | |
| *Synechocystis* sp. PAK13 | 0 | 2.891 ± 0.061 | 6.673 ± 0.158 | 0.817 ± 0.017 | 5.372 ± 0.124 | 1.648 ± 0.043 | 0.144 ± 0.004 | 17.544 ± 0.407 |
| | 1.66 | 1.560 ± 0.041* | 5.712 ± 0.115* | 1.843 ± 0.042** | 2.756 ± 0.051** | 1.845 ± 0.048** | 0.359 ± 0.009** | 14.075 ± 0.307 |
| | 3.33 | 1.766 ± 0.033* | 6.543 ± 0.145ns | 1.757 ± 0.033** | 2.941 ± 0.056** | 2.432 ± 0.063** | 0.242 ± 0.006** | 15.682 ± 0.337 |
| | 6.66 | 2.005 ± 0.037** | 9.991 ± 0.238** | 1.149 ± 0.024** | 2.438 ± 0.047** | 2.897 ± 0.075** | 0.198 ± 0.005** | 18.677 ± 0.427 |
| | 13.33 | 2.534 ± 0.047* | 12.755 ± 0.305** | 1.672 ± 0.032** | 1.326 ± 0.029** | 3.872 ± 0.101** | 0.243 ± 0.006** | 22.403 ± 0.520 |
| | 26.66 | 3.375 ± 0.067** | 17.474 ± 0.429** | 0.935 ± 0.026ns | 2.734 ± 0.052** | 2.158 ± 0.056** | 0.229 ± 0.006** | 26.906 ± 0.636 |
| *Chlorella variabilis* DT025 | 0 | 2.119 ± 0.040 | 8.493 ± 0.192 | 0.684 ± 0.062 | 1.108 ± 0.032 | 3.530 ± 0.123 | 0.277 ± 0.007 | 16.212 ± 0.456 |
| | 1.66 | 2.219 ± 0.043 ns | 11.178 ± 0.273** | 0.611 ± 0.019* | 2.751 ± 0.056** | 1.461 ± 0.038* | 0.162 ± 0.004** | 18.381 ± 0.433 |
| | 3.33 | 2.159 ± 0.042 ns | 14.489 ± 1.363ns | 2.221 ± 0.043** | 2.657 ± 0.049** | 2.895 ± 0.075* | 0.332 ± 0.009** | 24.753 ± 1.581 |
| | 6.66 | 2.421 ± 0.051 ns | 13.759 ± 0.344** | 2.080 ± 0.014** | 1.462 ± 0.028ns | 1.380 ± 0.036* | 0.121 ± 0.003** | 21.223 ± 0.477 |
| | 13.33 | 1.691 ± 0.031 ** | 15.353 ± 0.376** | 1.552 ± 0.097ns | 2.236 ± 0.042** | 2.656 ± 0.176ns | 0.208 ± 0.005** | 23.696 ± 0.728 |
| | 26.66 | 1.977 ± 0.037** | 11.001 ± 0.846ns | 1.814 ± 0.114** | 3.084 ± 0.059** | 3.105 ± 0.206 ns | 0.243 ± 0.006** | 21.224 ± 1.268 |

Data are presented as average of three independent replicates ± SE. The statistical significances $P > 0.05$, $P \leq 0.05$, $P \leq 0.01$, *and* $P \leq 0.001$ were marked by the symbols ns, * and **, respectively.

Table 2. Nonpolar amino acids content (mg/g) in *Synechocystis* sp. and *Chlorella* sp. grown under different concentrations of glycine for 14 days.

| Strain | Glycine Dose | Glycine | Alanine | Isoleucine | Leucine | Methionine | Valine | Phenylalanine | Total nonpolar amino acids |
|---|---|---|---|---|---|---|---|---|---|
| | | Mean ± SE | Mean ± SE | Mean ± SE | Mean ± SE | Mean ± SE | Mean ± SE | Mean ± SE | |
| *Synechocystis* sp. PAK13 | 0 | 21.148 ± 0.336 | 5.564 ± 0.081 | 1.775 ± 0.020 | 0.249 ± 0.020 | 0.311 ± 0.062 | 0.382 ± 0.010 | 0.667 ± 0.128 | 30.096 ± 0.656 |
| | 1.66 | 43.778 ± 0.633**** | 6.846 ± 0.102**** | 0.224 ± 0.034**** | 0.318 ± 0.026** | 0.197 ± 0.003ns | 0.671 ± 0.015 ns | 0.524 ± 0.110 ns | 52.558 ± 0.922 |
| | 3.33 | 52.060 ± 0.604*** | 3.992 ± 0.047** | 0.094 ± 0.001**** | 0.023 ± 0.002*** | 0.034 ± 0.008ns | 0.337 ± 0.011 ns | 0.531 ± 0.006 ns | 57.072 ± 0.680 |
| | 6.66 | 73.049 ± 1.498**** | 11.622 ± 0.152** | 0.174 ± 0.006**** | 0.078 ± 0.006**** | 0.069 ± 0.007* | 0.633 ± 0.018 ns | 0.911 ± 0.130 ns | 86.536 ± 1.818 |
| | 13.33 | 72.659 ± 8.821*** | 6.819 ± 0.118*** | 1.355 ± 0.020ns | 0.106 ± 0.009*** | 0.119 ± 0.020 ns | 0.733 ± 0.088 ns | 0.839 ± 0.082 ns | 82.632 ± 9.158 |
| | 26.66 | 125.408 ± 4.798*** | 16.982 ± 0.321**** | 0.109 ± 0.001**** | 0.018 ± 0.001**** | 0.034 ± 0.010 ns | 0.914 ± 0.029 ns | 1.012 ± 0.098 ns | 144.477 ± 5.259 |
| *Chlorella variabilis* DT025 | 0 | 25.650 ± 0.585 | 9.754 ± 0.161 | 0.135 ± 0.001 | 0.021 ± 0.002 | 0.043 ± 0.014 | 0.932 ± 0.013 | 0.376 ± 0.097 | 36.911 ± 0.873 |
| | 1.66 | 35.090 ± 0.433*** | 6.780 ± 0.093** | 0.152 ± 0.019ns | 0.186 ± 0.015* | 0.239 ± 0.049ns | 0.713 ± 0.051 ns | 1.311 ± 0.113 ** | 44.471 ± 0.774 |
| | 3.33 | 46.817 ± 0.515**** | 10.860 ± 0.211* | 0.151 ± 0.006ns | 0.071 ± 0.006ns | 0.065 ± 0.006ns | 0.818 ± 0.014 ns | 0.548 ± 0.251 ns | 59.328 ± 1.007 |
| | 6.66 | 53.903 ± 1.421** | 16.193 ± 0.322*** | 0.129 ± 0.011ns | 0.118 ± 0.010ns | 0.122 ± 0.016ns | 1.098 ± 0.012 ns | 0.788 ± 0.151 ns | 72.352 ± 1.943 |
| | 13.33 | 61.824 ± 0.866*** | 15.204 ± 0.295** | 0.211 ± 0.008ns | 0.099 ± 0.009* | 0.090 ± 0.008ns | 1.145 ± 0.019 ns | 1.098 ± 0.169 * | 79.672 ± 1.373 |
| | 26.66 | 69.300 ± 3.006* | 7.478 ± 0.109** | 2.140 ± 0.024**** | 0.317 ± 0.027** | 0.404 ± 0.076ns | 0.751 ± 0.028 ns | 0.810 ± 0.165 ns | 81.200 ± 3.437 |

Data are presented as average of three independent replicates ± SE. The statistical significances $P > 0.05$, $P \leq 0.05$, $P \leq 0.01$, $P \leq 0.001$ *and* $P \leq 0.0001$ were marked by the symbols ns, *, ** and ***, ****, respectively.

Table 3. Detection different polar amino acids content (mg/g) in *Synechocystis* sp. and *Chlorella* sp. exposure of different concentrations of glycine for 14 days.

| Strain | Glycine Dose | Glutamine | Asparagine | Threonine | Serine | Cystine | Tyrosine | Total polar amino acids |
|---|---|---|---|---|---|---|---|---|
| | | Mean ± SE | Mean ± SE | Mean ± SE | Mean ± SE | Mean ± SE | Mean ± SE | |
| *Synechocystis* sp. PAK13 | 0 | 2.655 ± 0.622 | 4.950 ± 0.189 | 0.565 ± 0.044 | 0.222 ± 0.050 | 0.388 ± 0.098 | 0.652 ± 0.014 | 9.433 ± 1.017 |
| | 1.66 | 0.324 ± 0.006$^{****}$ | 0.121 ± 0.019$^{****}$ | 0.106 ± 0.092$^{ns}$ | 0.257 ± 0.035$^{ns}$ | 0.102 ± 0.086$^{ns}$ | 0.559 ± 0.012$^{ns}$ | 1.468 ± 0.250 |
| | 3.33 | 0.685 ± 0.077$^{****}$ | 0.594 ± 0.007$^{****}$ | 0.154 ± 0.044$^{ns}$ | 0.276 ± 0.075$^{ns}$ | 0.727 ± 0.069$^{ns}$ | 0.507 ± 0.011$^{ns}$ | 2.943 ± 0.283 |
| | 6.66 | 0.747 ± 0.066$^{****}$ | 0.558 ± 0.015$^{****}$ | 0.208 ± 0.084$^{ns}$ | 0.381 ± 0.080$^{ns}$ | 0.070 ± 0.128$^{ns}$ | 0.828 ± 0.018$^{ns}$ | 2.793 ± 0.391 |
| | 13.33 | 3.970 ± 0.156$^{**}$ | 1.955 ± 0.178$^{****}$ | 0.312 ± 0.070$^{ns}$ | 0.878 ± 0.078$^{*}$ | 0.255 ± 0.063$^{ns}$ | 0.423 ± 0.009$^{ns}$ | 7.793 ± 0.555 |
| | 26.66 | 0.669 ± 0.098$^{****}$ | 0.731 ± 0.009$^{****}$ | 0.295 ± 0.120$^{ns}$ | 0.543 ± 0.103$^{ns}$ | 0.110 ± 0.062$^{ns}$ | 0.404 ± 0.009$^{ns}$ | 2.751 ± 0.401 |
| *Chlorella variabilis* DT025 | 0 | 2.391 ± 0.160 | 1.310 ± 0.015 | 0.352 ± 0.130 | 0.223 ± 0.020 | 0.157 ± 0.071 | 0.461 ± 0.010 | 4.894 ± 0.406 |
| | 1.66 | 2.988 ± 0.485$^{*}$ | 3.388 ± 0.062$^{****}$ | 0.555 ± 0.077$^{ns}$ | 0.764 ± 0.138$^{ns}$ | 0.148 ± 0.061$^{ns}$ | 0.397 ± 0.009$^{ns}$ | 8.240 ± 0.831 |
| | 3.33 | 0.900 ± 0.079$^{****}$ | 0.649 ± 0.011$^{ns}$ | 0.238 ± 0.114$^{ns}$ | 0.206 ± 0.014$^{ns}$ | 0.092 ± 0.172$^{ns}$ | 1.113 ± 0.025$^{ns}$ | 3.198 ± 0.414 |
| | 6.66 | 0.656 ± 0.110$^{****}$ | 0.803 ± 0.009$^{ns}$ | 0.424 ± 0.159$^{ns}$ | 0.260 ± 0.059$^{ns}$ | 0.074 ± 0.055$^{ns}$ | 0.360 ± 0.008$^{ns}$ | 2.577 ± 0.400 |
| | 13.33 | 0.643 ± 0.057$^{****}$ | 0.502 ± 0.020$^{*}$ | 0.333 ± 0.159$^{ns}$ | 0.460 ± 0.093$^{ns}$ | 0.066 ± 0.123$^{ns}$ | 0.795 ± 0.018$^{ns}$ | 2.798 ± 0.469 |
| | 26.66 | 2.284 ± 0.535$^{ns}$ | 4.507 ± 0.250$^{****}$ | 0.789 ± 0.088$^{ns}$ | 0.413 ± 0.055$^{ns}$ | 0.359 ± 0.094$^{ns}$ | 0.626 ± 0.014$^{ns}$ | 8.977 ± 1.036 |

Data are presented as average of three independent replicates ± SE. The statistical significances $P > 0.05$, $P \leq 0.05$, $P \leq 0.01$, $P \leq 0.001$ *and* $P \leq 0.0001$ were marked by the symbols $^{ns}$, *, ** and ***, ****, respectively.

Table 4. Basic and acidic amino acids content (mg/g) in *Synechocystis* sp. and *Chlorella* sp. exposure of different concentrations of glycine for 14 days.

| Strain | Glycine Dose | Basic or positive charge amino acids | | | Total basic amino acids | Acidic or negative charge amino acids | | Total acidic amino acids | Total amino acids |
|---|---|---|---|---|---|---|---|---|---|
| | | Lysine | Histidine | Arginine | | Glutamic acid | Aspartate | | |
| | | Mean ± SE | Mean ± SE | Mean ± SE | | Mean ± SE | Mean ± SE | | |
| *Synechocystis* sp. PAK13 | 0 | 2.135 ± 0.131 | 1.497 ± 0.135 | 1.101 ± 0.025 | 4.733 ± 0.291 | 0.924 ± 0.010 | 0.186 ± 0.022 | 1.110 ± 0.032 | 45.373 ± 1.997 |
| | 1.66 | 3.007 ± 0.095$^{**}$ | 1.090 ± 0.048$^{ns}$ | 0.481 ± 0.028$^{*}$ | 4.579 ± 0.170 | 0.791 ± 0.009$^{ns}$ | 0.102 ± 0.001$^{ns}$ | 0.893 ± 0.010 | 59.498 ± 1.353 |
| | 3.33 | 3.182 ± 0.115$^{***}$ | 1.229 ± 0.044$^{*}$ | 0.472 ± 0.034$^{*}$ | 4.883 ± 0.193 | 0.090 ± 0.008$^{**}$ | 0.211 ± 0.077$^{ns}$ | 0.301 ± 0.085 | 65.199 ± 1.241 |
| | 6.66 | 3.785 ± 0.114$^{****}$ | 1.499 ± 0.116$^{ns}$ | 0.983 ± 0.028$^{ns}$ | 6.266 ± 0.258 | 0.977 ± 0.012$^{ns}$ | 0.145 ± 0.003$^{ns}$ | 1.122 ± 0.015 | 96.717 ± 2.481 |
| | 13.33 | 1.097 ± 0.097$^{***}$ | 1.294 ± 0.198$^{*}$ | 1.444 ± 0.016$^{ns}$ | 3.834 ± 0.311 | 0.657 ± 0.010$^{ns}$ | 0.163 ± 0.014$^{ns}$ | 0.820 ± 0.024 | 95.078 ± 10.048 |
| | 26.66 | 3.282 ± 0.086$^{****}$ | 1.060 ± 0.049$^{*}$ | 0.564 ± 0.023$^{*}$ | 4.905 ± 0.158 | 0.785 ± 0.012$^{ns}$ | 0.149 ± 0.002$^{ns}$ | 0.934 ± 0.014 | 153.067 ± 5.831 |
| *Chlorella variabilis* DT025 | 0 | 4.063 ± 0.063 | 0.952 ± 0.053 | 0.397 ± 0.060 | 5.413 ± 0.176 | 0.687 ± 0.008 | 0.094 ± 0.004 | 0.782 ± 0.011 | 48.000 ± 1.466 |
| | 1.66 | 3.361 ± 0.108$^{ns}$ | 1.529 ± 0.160$^{ns}$ | 1.256 ± 0.022$^{*}$ | 6.146 ± 0.290 | 0.928 ± 0.015$^{ns}$ | 0.195 ± 0.003$^{ns}$ | 1.122 ± 0.018 | 59.980 ± 1.913 |
| | 3.33 | 6.122 ± 0.118$^{****}$ | 1.436 ± 0.032$^{ns}$ | 0.340 ± 0.047$^{ns}$ | 7.898 ± 0.197 | 1.723 ± 0.019$^{**}$ | 0.175 ± 0.004$^{ns}$ | 1.898 ± 0.023 | 72.322 ± 1.642 |
| | 6.66 | 4.089 ± 0.077$^{ns}$ | 0.195 ± 0.036$^{*}$ | 0.256 ± 0.003$^{ns}$ | 4.540 ± 0.116 | 1.092 ± 0.012$^{ns}$ | 0.145 ± 0.002$^{ns}$ | 1.236 ± 0.015 | 80.704 ± 2.474 |
| | 13.33 | 4.772 ± 0.215$^{*}$ | 2.010 ± 0.045$^{***}$ | 0.710 ± 0.079$^{ns}$ | 7.493 ± 0.338 | 1.257 ± 0.015$^{ns}$ | 0.180 ± 0.004$^{ns}$ | 1.437 ± 0.019 | 91.400 ± 2.199 |
| | 26.66 | 2.767 ± 0.235$^{****}$ | 2.381 ± 0.177$^{****}$ | 1.519 ± 0.046$^{***}$ | 6.667 ± 0.458 | 1.191 ± 0.013$^{ns}$ | 0.211 ± 0.019$^{ns}$ | 1.402 ± 0.032 | 98.246 ± 4.963 |

Data are presented as average of three independent replicates ± SE. The statistical significances $P > 0.05$, $P \leq 0.05$, $P \leq 0.01$, $P \leq 0.001$ *and* $P \leq 0.0001$ were marked by the symbols $^{ns}$, *, ** and ***, ****, respectively.

## **The results of 16S rRNA sequences for *Synechocystis* was:**

“ATTTTGCTACTAGGATTATCTTTCCTCCTACCTTCACCGCCCTTCAGTCTCACACTCA
GCCAAAAGCCTTGTGGTTAATATCAAAACGGTTAGTAAGAGCAAGATTTTCCCTGCT
TTCTCTTTTCTTTATGCAGTTTTCAAGGTCCTTACTGGACTTACATCCAGCATTCCCTC
TGACTCTCATCAGACAGGATGCTGAAGGATTTATCCTTCTCTTTGGCTTTTTTGGTTA
GGTGGGCCATTCTGGACTTGAACCAGAGACCTCACCCTTATCAGGGGTGCGCTCTAA
CCAGCTGAGCTAATAGCCCTTGCCTTTTACCTCGCCTAGGCAATAGTTTGAAAGACT
TCTACTCTTTAACCTCTCGGTTAATCCCTTGCTCGACCTTTTGGTTGACCAACTTCTCT
CTATTTGCACTTTGCTTTCAAGATGAAGGGGTAAAGGTCTCCCTTAAAGGAGGTGAT
CCAGCCACACCTTCCGGTACGGCTACCTTGTTACGACTTCACCCCAGTCACTAGCCC
TGCCTTCGGCGCCCTCCTCCCTAAGGTTAGAGTAACGACTTCGGGGGTGAGTCGTAC
AAGGTAGCCGTACCGGAAGGTGTGGCTGGATCACCTCCTTTAAGGGAGACCTTTACC
CCTTCATCTTGAAAGCAAAGTGCAAATAGAGAGAAGTTGGTCAACCAAAAGGTCGA
GCAAGGGATTAACCGAGAGGTTAAAAAGTATAAGTCTTTCAAACTATTGCCTAGGCT
AGGTAAAAGGCAAGGGCTATTAGCTCAGCTGGTTAGAGCGCACCCCTGATAAGGGT
GAGGTCTCTGGTTCAAGTCCAGAATGGCCCACCTAACCAAAAAAGCCAAAGAGAAG
GATAAATCCTTCAGCATCCTGTCTGATGAGAGTCAGAGGGAATGCTGGATGTAAGTC
CAGTAAGGACCTTGAAAACTGCATAAAGAAAAGAGAAAGCAGGGAAAATCTTGCTC
TTACTAACCGTTTTGATATTAACCACAAGGCTTTTGGCTGAGTGTGAGACTGAAGGG
CGGTGAAGGTAGGAGGAAAGATAATCCTAGTTAGCAAAAAGAAGATCTTAAA
AAGGTCAAGATACAAAGGGCTAACGGTGGATACCTAGCCCACA”

**<u>Figure 1. The phylogenetic tree of 16S rRNA sequences for *Synechocystis* was:</u>**

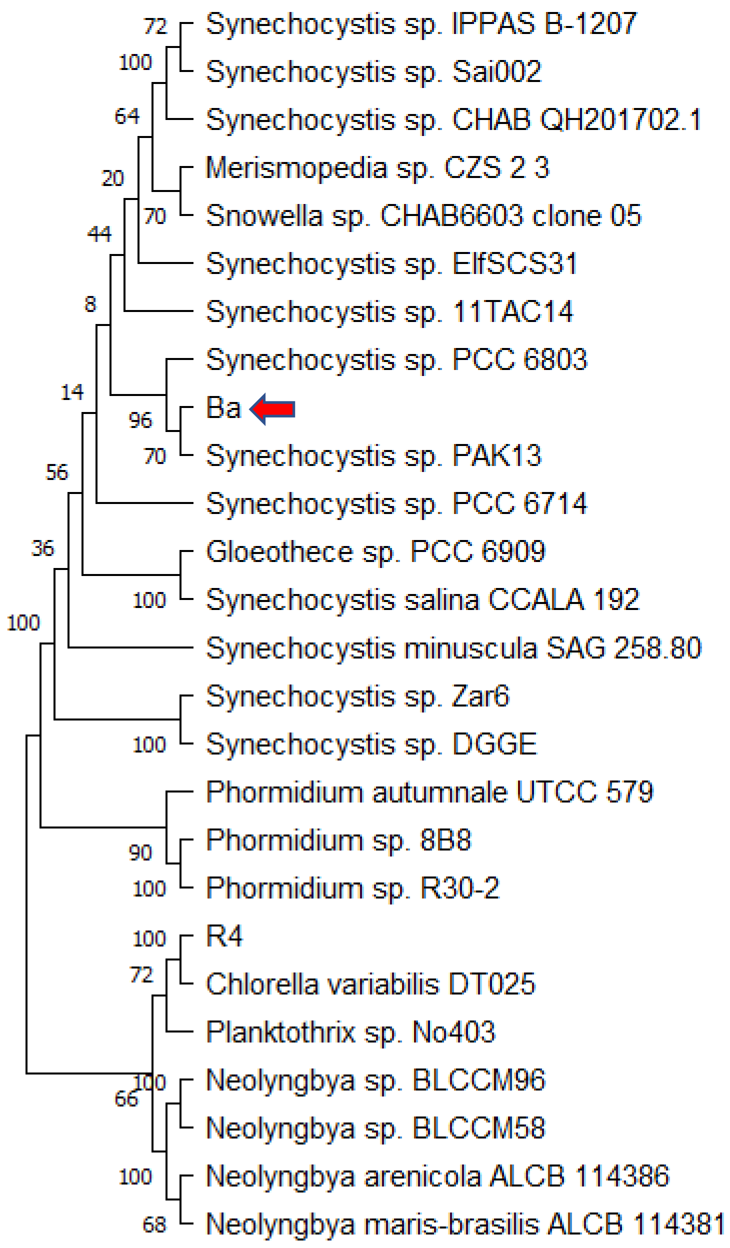

**<u>The results of 18S rRNA sequences for *Chlorella* was:</u>**

“CAAGGTAGCCGTACTGGAAGGTGCGGCTGGAACACCTCCTTTAAAAGGATACTTAT
ATTGTCTTCAAATCTCTATTTAAGGAGTCCGAAGAACCTTTTCCAAAAATTCTTCGAA
ATTTTAGGGAAAAAGTAAAATCCAATTTTACTGTATCGTTTTTTTGCTGTGCATGAGC
CACTAGCTCGCTTTTTTTCAAACCATTCCTTCAAATTGCACGCAATTTGAAGGACCAA
AAGTTAACAACATTATTGTGTTGGGTAAGGTCTTTTGTATAAAAAAGCCCAAGCAAC
GGGCTATTAGCTCAGTTGGTTAGAGCGCACCCCTGATAAGGGTGAGGTCGCTGGTTC
AAATCCAGCATAGCCCACCACACCATAAAAAACTTATGGGGGTATAGCTCAGTTGG
TAGAGCGCTGCCCTTGCAAGGCAGATGTCAGCGGTTCGAGTCCGCTTATCTCCACCA
GTTTTTTATTTTCGGCTTAGATTGTAAATAACTAAAAAAAGCAAGCTAGTGGCTCAT
GCACGGGCTCTCATTTCATTGTCTTTTTTGCTTGCAAAAAAGATACCAATATTGAAA
AATTTTTGGAGAGTGTAGTAGCCCCAAAAAACGTACATGGACCAAAAACGTCCACT
TTTTTTAACTTTAAAAAAAGTGGACGTTTTTGGTCCATGTACGTTTTTTGGGGCTACT
ACACTCTCCAAAAATTTTTCAATATTGGTATCTTTTTTGCAAGCAAAAAAGACAATG
AAATGAGAGCCCGTGCATGAGCCACTAGCTTGCTTTTTTTAGTTATTTACAATCTAA
GCCGAAAATAAAAAACTGGTGGAGATAAGCGGACTCGAACCGCTGACATCTGCCTT
GCAAGGGCAGCGCTCTACCAACTGAGCTATACCCCCATAAGTTTTTTATGGTGTGGT
GGGCTATGCTGGATTTGAACCAGCGACCTCACCCTTATCAGGGGTGCGCTCTAACCA
ACTGAGCTAATAGCCCGTTGCTTGGGCTTTTTTATACAAAAGACCTTACCCAACACA
ATAATGTTGTTAACTTTTGGTCCTTCAAATTGCGTGCAATTTGAAGGAATGGTTTGAA
AAAAAGCGAGCTAGTGGCTCATGCACAGCAAAAAAACGATACAGTAAAATTGGATT
TACTTTTTCCCTAAAATTTCGAAGAATTTTTGGAAAAGGTTCTTCGGACTCCTTAAAT
AGAGATTTGAAGACAATATAAGTATCCTTTTAAAGGAGGTGTTCCAGCCGCACCTTC
CAGTACGGCTACCTTGTTACGACTTCACCCCAGTCACTAGCTCTGCCTTAGGCGCCC
TCCTCCTTT”

**Figure 2. The phylogenetic tree of 18S rRNA sequences for *Chlorella* was:**

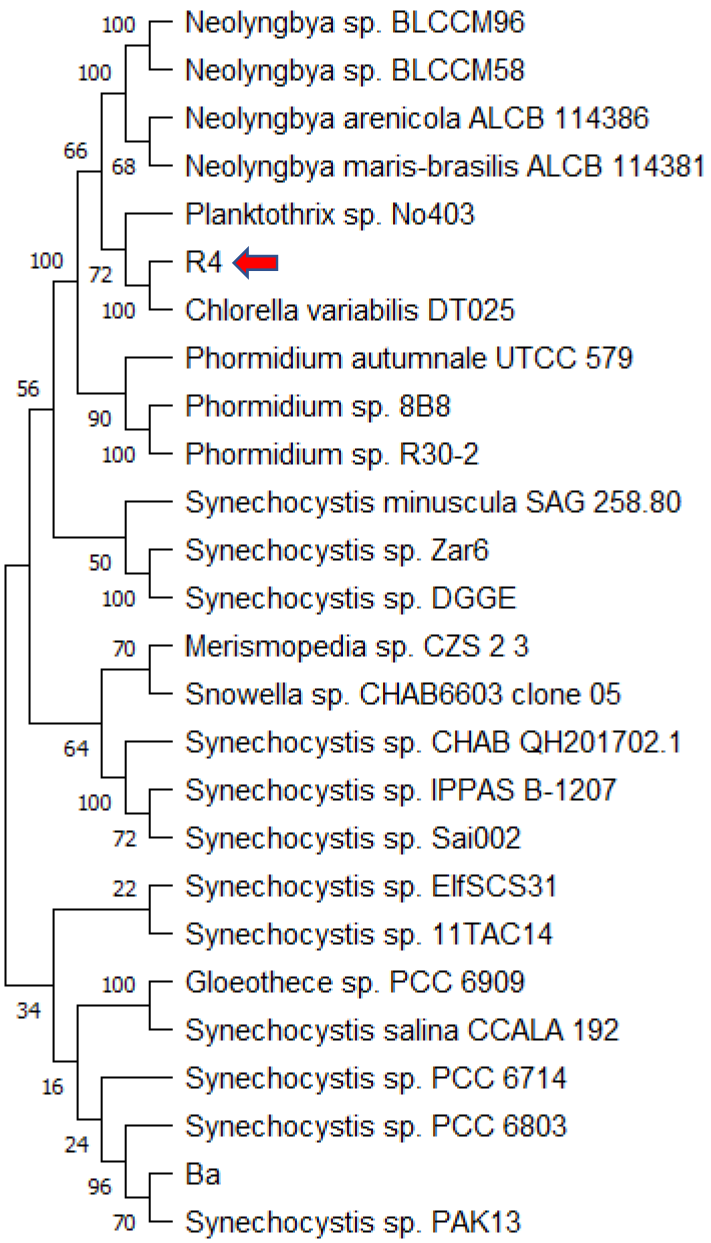

The micrograph of some in vivo *Synechocystis* sp. after one week the control sample first one in the left after that treated samples from 1.66, 3.33, 6.66, 13.33-, and 26.66-mM glycine:

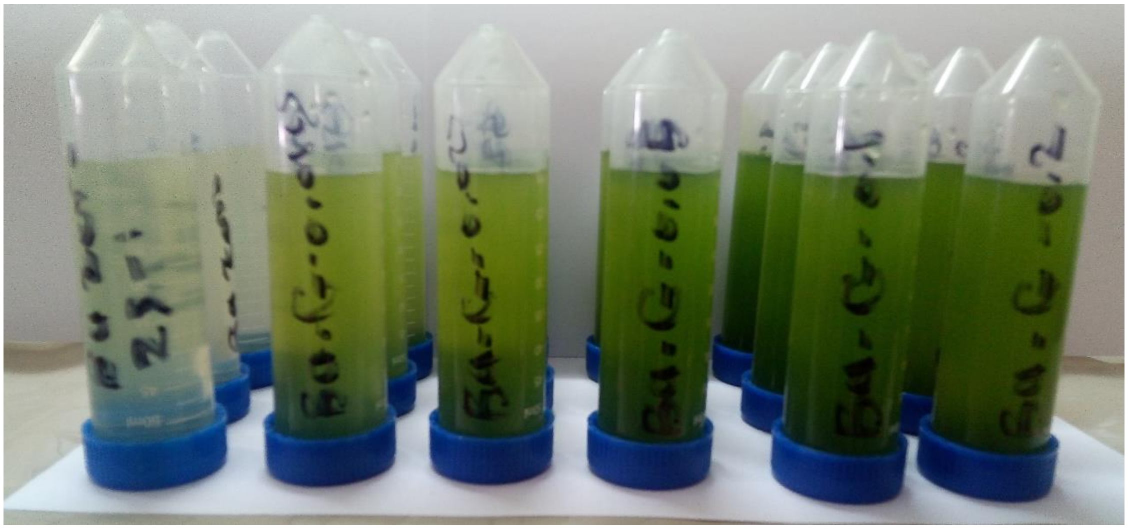

The micrograph of semi large scale of *Synechocystis* sp. treated with 26.66 mM glycine at the beginning of the culture:

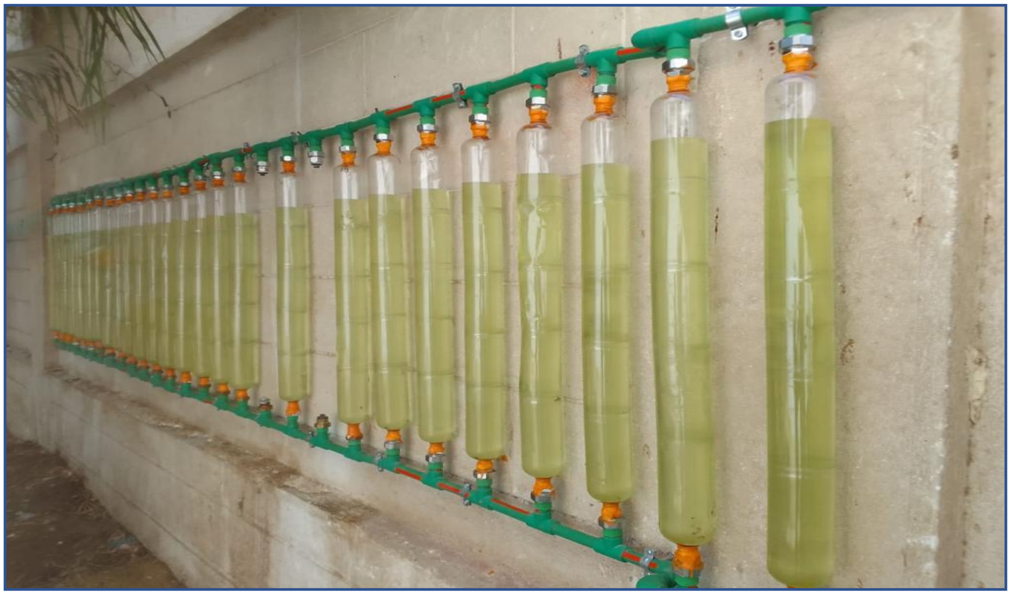

The micrograph of semi large scale of *Synechocystis* sp.  after two weeks treated with 26.66 mM glycine:

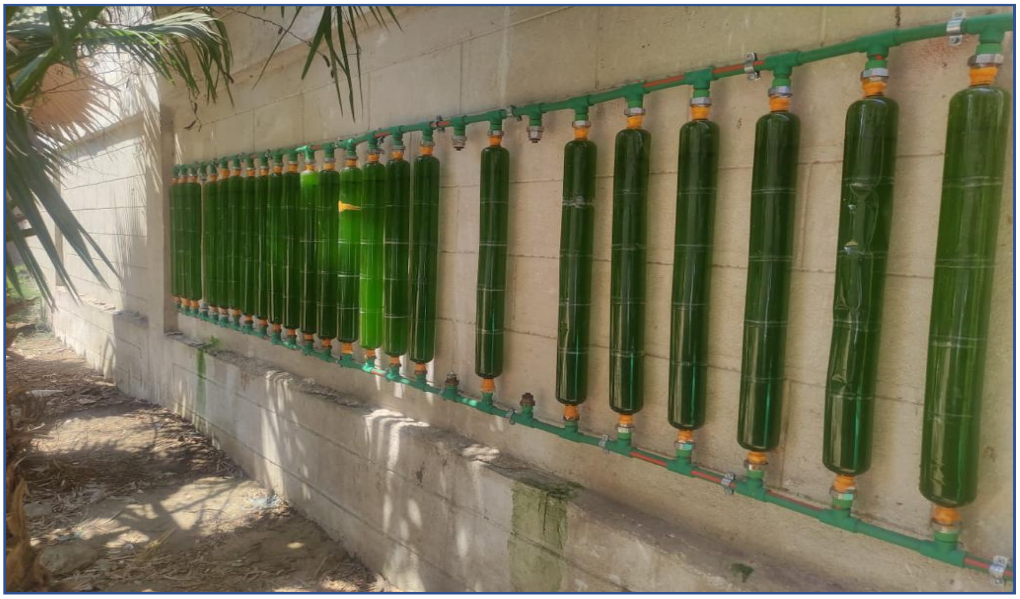

Supplement: Supplementary file 1 [file DataSheet1.PDF]
